# Immune Cytolytic Activity Correlates with Tumor Microenvironmental Aberrations in Colorectal Cancer

**DOI:** 10.3390/ijms27146180

**Published:** 2026-07-10

**Authors:** Stephanie Agioti, George Georgoulias, Ilias Georgakopoulos-Soares, Maria-Ioanna Christodoulou, Apostolos Zaravinos

**Affiliations:** 1 Cancer Genetics, Genomics and Systems Biology Laboratory, Basic and Translational Cancer Research Center (BTCRC), European University Cyprus, 1516 Nicosia, Cyprus; sa231751@students.euc.ac.cy (S.A.); gg161380@students.euc.ac.cy (G.G.); 2 Division of Pharmacology and Toxicology, College of Pharmacy, Dell Paediatric Research Institute, The University of Texas at Austin, Austin, TX 78712, USA; ilias@austin.utexas.edu; 3 Tumor Immunology and Biomarkers Laboratory, Basic and Translational Cancer Research Center (BTCRC), European University Cyprus, 1516 Nicosia, Cyprus; mar.christodoulou@euc.ac.cy

**Keywords:** colorectal cancer, immune cytolytic activity, tumor immune microenvironment, immune signatures, gene expression profiling, cancer neoepitopes, gene mutations, somatic copy number alterations, immune checkpoints, chromothripsis, LDH release assay, HCT-116, HT-29 colorectal cancer cell lines, 3D tumor models, immune cell infiltration, CD8^+^ T cells, apoptosis, GZMA, PRF1

## Abstract

Colorectal cancer (CRC) exhibits a highly heterogeneous tumor immune microenvironment (TME), ranging from “immune-inflamed” to “immune-desert” or “immune-excluded” phenotypes. Understanding how immune cell composition, cytolytic activity (CYT) and genomic alternations shape tumor-immune interactions is critical for improving immunotherapy outcomes. We analyzed TCGA-COAD and TCGA-READ datasets to evaluate immune competency, CYT, immune subtypes, microsatellite instability (MSI), and genomic instability, including somatic mutations, copy number aberrations (CNAs), and chromothriptic events. Immune cell infiltration was correlated with CYT levels, immune checkpoint expression, and immune-related gene signatures. Immune-competent (IC) tumors were predominantly CYT-high, enriched in stromal and immune scores, and exhibited distinct TME characteristics compared with immune-deficient (ID) tumors. IC/CYT-high tumors expressed higher levels of immune checkpoints (PD-1, PD-L1, CTLA-4, IDO1/2, LAG-3) and cytokines/chemokines (C1QA/B/C, CXCL9/10/11, CXCL13). Differences in immune infiltration were observed across tumors with significant mutations and copy number alterations. No prognostic difference was observed between CYT-high and CYT-low patients, indicating that CYT reflects immune activation rather than clinical outcome. Functionally, stimulated CD8^+^ T cells exhibited cytotoxicity activity against MSI-high (HCT-116) and microsatellite-stable (HT-29) CRC cells, with MSI-H cells showing higher sensitivity. Dynamic 3D co-culture demonstrated tumor-guided T cell infiltration and retention of CD8 expression, and co-culture was associated with moderate upregulation of cytotoxicity-related genes GZMA and PRF1 within the system. Cytotoxic activity decreased at lower effector-to-target ratios, highlighting the importance of effector dose. Overall, these findings link CYT, immune competency, MSI status, and genomic instability to T cell cytotoxic responses, providing insights into tumor-immune interactions, and suggest potential associations relevant for immunotherapy research in CRC.

## 1. Introduction

Colorectal cancer (CRC) is the third most common type of cancer and the second most deadly [1], with a prognosis that mainly depends on its stage at the time of diagnosis. It is among the most heterogeneous cancers with four distinct consensus molecular subtypes [2]. It is mainly characterized by genetic instability due to either chromosomal instability (CIN) [3] or deficiency in the DNA mismatch repair (dMMR) system [4], which leads to high microsatellite instability (MSI-H), usually with MLH1 silencing, and the accumulation of further mutations [5,6]. Such DNA lesions, once accumulated will often generate immunogenic cancer neoepitopes, which will attract a high number of inflammatory cell types into the tumor microenvironment (TME), eventually contributing to its pathogenesis [7].

Although the standard treatment option includes surgical removal usually followed by adjuvant 5-FU chemotherapy and/or targeted therapy depending on the tumor’s stage [8], over the past decade, immunotherapy has enabled never-before-seen success rates in durable tumor control and enhanced survival benefit in patients with advanced cancers. Importantly, immune checkpoint inhibition (ICI) therapies using specific anti-PD-1/PD-L1 and/or anti-CTLA-4 monoclonal antibodies to treat metastatic patients with dMMR/MSI-H have spawned overwhelming enthusiasm for immunotherapy in the disease [9,10]. However, the dMMR/MSI-H metastatic patients are just a fraction of the total CRC patient population, and the vast majority of them having a proficient MMR system or absence of microsatellite instability (pMMR/MSS) will not benefit from such ICI therapies.

Deficiency in the DNA mismatch repair (dMMR) system is associated with increased genomic instability and distinct mutational processes in colorectal cancer [11]. Among these, chromothripsis represents a catastrophic genomic event characterized by extensive chromosome fragmentation and reassembly occurring in a single crisis. Chromothriptic events have been reported across multiple tumor types, including colorectal cancer, and are associated with complex structural alterations that may influence tumor evolution and immune interactions [12,13].

Tumors with chromothripsis were associated with reduced infiltration of CD8^+^ T cells, diminished antitumor immune responses, and a weaker response to ICI immunotherapy, whereas tumors without chromothripsis were associated with longer survival following immunotherapy [14].

Copy number aberrations (CNAs) are frequently observed in MSS colon tumors and are associated with the upregulation of gene expression profiles and their signaling pathways, which promote carcinogenesis [15]. An increased copy number load is associated with low antitumor immune activity, immune evasion, and resistance to immunotherapy [16].

The classification for different immunological subtypes in CRC can predict patient response to ICI-therapies and enhance antitumor activity [17] and current efforts focus on how we can reprogram the TME to improve such approaches [18]. The TME is the ecosystem surrounding a tumor and includes the extracellular matrix, fibroblasts, blood vessels and stromal cells. It also encompasses different types of immune cells, including neutrophils, dendritic cells (DCs), natural killer (NK) cells, T cells and B cells, myeloid-derived suppressor cells (MDSCs), and tumor-associated macrophages (TAMs). Its composition may predict patient prognosis and response to ICI therapies [19,20].

One of the TME’s crucial components are tumor-infiltrating lymphocytes (TILs) which enhance the anti-tumor immune responses by recognize and kill autologous cancer cells [21]. They are mainly composed of CD8^+^/CD4^+^ T cells [22] and are currently one of the most promising types of cancer immunotherapy due to their high efficacy [23]. As the TME significantly contributes to cancer progression [24], and abnormalities in it can interrupt immunotherapeutic approaches, its better understanding should provide new insights into the immunotherapy of the disease. In addition, the heterogeneity of the TME has not been evaluated in-depth.

Cytotoxic T lymphocytes (CTLs) can target and kill cancer cells through the granzyme-perforin pathway [25]. Perforins open pores in the membrane of the targeted cancer cells, facilitating the entry of granzymes in them, which eventually eliminate the cancer cell in an apoptotic manner [25,26]. The intratumoral immune cytolytic activity (CYT) is a relatively new measure of immune activation, based on the geometric mean of the expression of the above-mentioned toxins [25,27]. The eliminated cancer cells release cancer neoepitopes that further stimulate an adaptive immunity. Nevertheless, cancer cells have developed various mechanisms to escape immune surveillance, such as by secreting soluble cytokines or chemokines, which can recruit suppressive cells [e.g., T-regulatory cells (Tregs) and MDSCs] in the TME, or by regulating T-cell activation through co-stimulatory and co-inhibitory signals (e.g., PD-1, CD86/CD80, CTLA-4, etc.) [28], leading to an immunosuppressive TME [29].

As the incidence of CRC is increasing, and most patients with CRC, especially those with pMMR/MSS CRC tumors still have a poor prognosis or develop resistance to ICI immunotherapy, we aimed to investigate the TME in CRC patients using transcriptomics and genomics analyses. Specifically, we sought to identify two immune subtypes of CRC tumors associated with the patients’ CYT levels and to elucidate their immunological and microenvironmental characteristics. In the current study we aimed to identify the mechanisms responsible for aberrations within the TME and to understand how immune cells and CYT associate with these aberrations in order to identify new therapeutic targets or potential biomarkers, ultimately improving disease management, enhancing quality of life, and increasing the lifespan of patients.

While the association between MSI-H status and elevated immune cytolytic activity in colorectal cancer has been previously described [17,27], here we extend these findings by integrating CYT with immune competency status, large-scale genomic instability features including chromothripsis, and functional tumor-immune interactions assessed using a dynamic 3D co-culture system. To our knowledge, this is the first study to descriptively link chromothriptic events with CYT-defined immune states in CRC and to combine immunogenomic stratification with real-time immune cell extravasation and cytotoxicity assays.

## 2. Results

### 2.1. Immune-Competent CRC Tumors Are Mainly CYT-High and Demonstrate Differential TME Characteristics from the Immune-Deficient Ones

**Figure 1:**
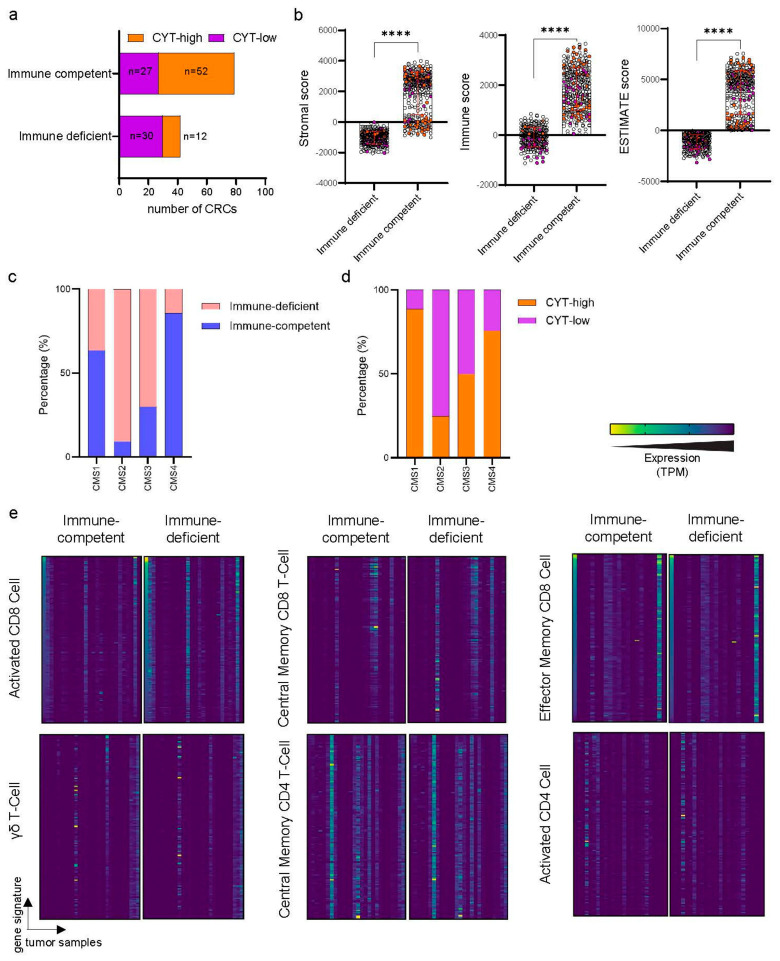
(**a**) The majority of immune-competent (IC, 52/79) CRCs were CYT-high and that of immune-deficient (ID) tumors was CYT-low (30/42). (**b**) Scatter dot-plots depict the stromal, immune and tumor purity (ESTIMATE) scores. IC/ID classification reflects ESTIMATE-derived immune contexture. The mean values with standard deviation (SD) are depicted in red color. Orange circles denote CYT-high tumors, while purple ones denote CYT-low. Uncolored dots are CYT-intermediate. Statistically significant differences are noted with stars (****, *p* < 0.00001). (**c**) Distribution of immune-competent (IC) vs. immune-deficient (ID) tumors across the different consensus molecular subtypes (CMS1–4). (**d**) Distribution of CYT-high vs. CYT-low tumors across the different CMS subtypes. (**e**) Viridis colormaps depict various immune-related gene signatures in IC and ID tumors. Tumor samples are shown along the *x*-axis, while genes comprising each immune signature are displayed along the *y*-axis. Color intensity represents normalized gene expression values (log_2_[TPM + 1]), with darker colors indicating higher expression. Panels correspond to selected immune cell signatures relevant to cytotoxic and adaptive immune responses. Additional immune signatures are provided in Supplementary Figure S3.

Comparing immune-competency and cytolytic levels among tumors, we found a high match between immune-competent (IC) and CYT-high CRCs (65.8%, 52/79), while 71.4% (30/42) of immune-deficient (ID) tumors were at the same time CYT-low (Figure 1a). Notably, the proportion of stage IV disease was comparable between CYT-high and CYT-low tumors (17.5% vs. 16.7%, respectively), indicating that elevated cytolytic activity does not prevent metastatic progression (Supplementary Table S3). Age-stratified descriptive revealed that both CYT-high and CYT-low tumors, as well as IC and ID subgroups, were predominantly diagnosed in patients ≥50 years of age, with no apparent enrichment of specific immune phenotypes in early-onset CRC (<50 years) (Supplementary Figure S1). As expected from the definition of the IC/ID classification, IC/CYT-high tumors exhibited higher ESTIMATE immune and stromal scores, reflecting their classification based on ESTIMATE-derived metrics (Figure 1b).

Application of the consensus molecular subtype (CMS) classification to the TCGA-COAD cohort revealed heterogeneous distribution across the four subtypes, with CMS2 representing the largest proportion of tumors (n = 304), followed by CMS4 (n = 95), CMS1 (n = 77), and CMS3 (n = 48). Integration with clinical annotations demonstrated a strong enrichment of microsatellite instability (MSI) within CMS1 tumors, whereas CMS2, CMS3, and CMS4 were predominantly microsatellite stable (MSS). Investigating whether the consensus molecular subtype impacts the cytolytic status or immune competency in each tumor, we found that CMS1 and CMS4 colon adenocarcinomas are dominated by immune-competent and CYT-high tumors; whereas CMS2 tumors are largely immune-deficient and CYT-low. CMS3 tumors are more mixed, with noticeable spread across categories (Figure 1c,d and Supplementary Figure S2).

To better explore the microenvironmental features in IC tumors, we further compared the estimated abundance of infiltrated immune cells within them. As expected, our analysis revealed that most of the immune cells examined are significantly infiltrated in the TME of IC CRCs. Several immune signatures (e.g., γδ T-cell, effector memory CD4 T-cell, central memory CD8 T-cell, regulatory T-cell, among others), were significantly enriched in IC tumors. On the other hand, most of the examined immune cells were expressed at lower levels in ID tumors. In addition, expression differences in several immune signatures were driven by subsets of immune-enriched tumors rather than uniform changes across all samples, consistent with the heterogeneous immune landscape of colorectal cancer (Figure 1e).

### 2.2. Immune-Related Gene Expression Signatures Across CYT and Immune-Competency Subgroups of CRC Tumors

As the IC tumors demonstrated relatively higher abundance of infiltrated immune cells, we further evaluated the expression of signature genes for macrophages, activated CD4^+^ and CD8^+^ T cells, T regulatory cells (Tregs), NK cells, and neutrophils in them and evaluated differences between cytolytic and immune-competent/deficient subgroups. Of note, we found differences in immune-related gene expression signatures between CYT-high and CYT-low tumors. Specifically, all immune cell-specific signatures were higher in CYT-high CRCs compared to CYT-low ones.

**Figure 2:**
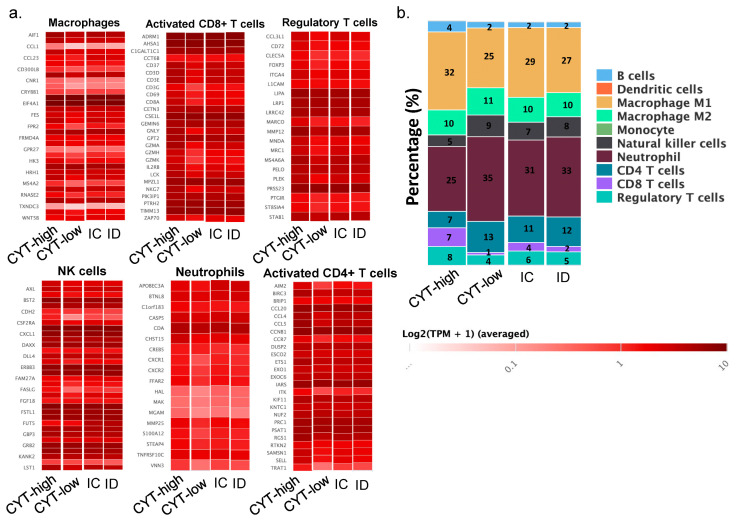
(**a**) Expression signatures for macrophages, activated CD4^+^ / CD8^+^ T cells, Tregs, NK cells and neutrophils in CYT-high, CYT-low, immune competent (IC) and immune deficient (ID) CRC tumors. (**b**) Immune cell-type fraction analysis in CYT-high, CYT-low, IC and ID CRCs.

On the other hand, we did not find any difference in the expression of immune-related genes between the IC and ID subgroups of tumors (Figure 2a).

### 2.3. Cell Type Fraction Analysis Across CYT and Immune Competency Subgroups of Tumors

As our results suggested that IC tumors have a relatively higher abundance of infiltrated immune cells compared to the ID ones, we further measured the cell type fractions both per cytolytic subgroup, as well as according to the immune competency status of the tumors. Overall, CYT-high tumors exhibited a similar fraction of immune cells to that of IC tumors, and CYT-low tumors with the ID ones. In specific, IC tumors contained a slightly higher number of M1 macrophages vs. ID (29% vs. 27%), CD8^+^ T cells (4% vs. 2%) and Tregs (6% vs. 5%); while ID tumors were enriched in neutrophils (33%), NK cells (8%) and CD4^+^ T cells (12%). Both IC and ID tumors contain 2% of B cells, 10% of M2 macrophages and <0.5% of DCs. On the other hand, CYT-low tumors contained higher number of NK cells (9%), neutrophils (35%) and activated CD4^+^ T cells (13%), while CYT-high CRCs were enriched in M1 macrophages (32%), CD8^+^ T cells (7%) and Tregs (8%). Both cytolytic subgroups contained 0% of monocytes and DCs (Figure 2b).

### 2.4. Correlation of the Expression of Immune Checkpoints, Cytokines and Immune-Related Genes with the Level of Immune Infiltration in CRC

**Figure 3:**
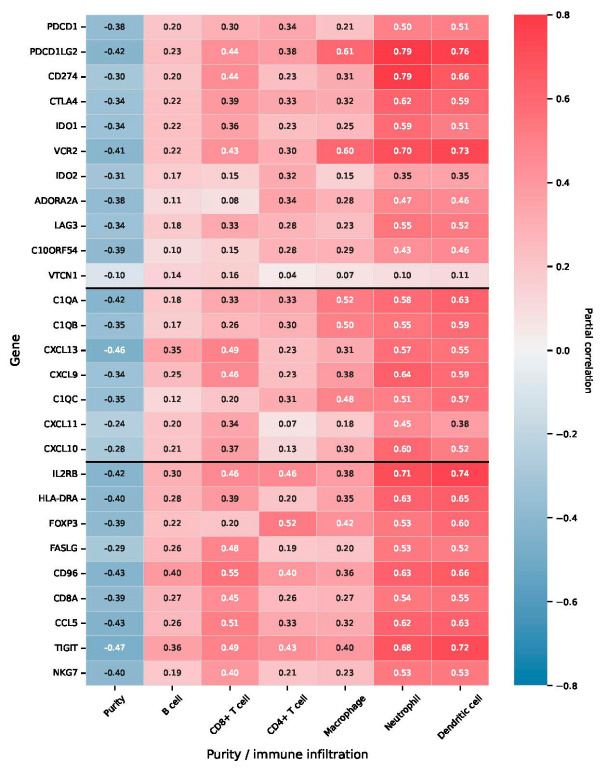
Partial correlation heatmap between immune-related genes and tumor purity or immune cell infiltration in colorectal cancer. The heatmap summarizes partial Spearman correlation coefficients between the expression of immune checkpoint genes, cytokines, and immune-related genes (rows) and tumor purity or estimated immune cell infiltration levels (columns), including B cells, CD8^+^ T cells, CD4^+^ T cells, macrophages, neutrophils, and dendritic cells. Correlations were calculated using TIMER after adjustment for tumor purity. Color intensity reflects the direction and magnitude of the partial correlation (blue, negative; red, positive), as indicated by the scale bar. Tumor purity shows predominantly negative correlations with immune-related gene expression, whereas immune cell infiltration levels exhibit predominantly positive correlations, consistent with immune-enriched tumor microenvironments. Only correlations meeting the predefined significance threshold are displayed.

We then investigated whether high checkpoint expression is linked to reduced immune cell activity, a key feature of immune suppression, by analyzing the relationship between immune checkpoint expression and the infiltration levels of different immune cells. Our analysis showed that overexpression of *PDCD1 (PD-1)*, *CD274 (PD-L1)*, *PDCD1LG2 (PDL2)*, *CTLA-4*, *IDO1*, *IDO2*, *HAVCR2 (TIM3)*, *ADORA2A (A2AR)*, *LAG3*, *VISTA (C10orf54)* and *VTCN1* (*B7-H4*) is positively correlated with the infiltration of B cells, CD8^+^ T cells, CD4^+^ T cells, macrophages, neutrophils and DCs in COAD. On the contrary, their over-expression was negatively correlated with the tumor’s purity, reflecting the low tumor purity and thus, the high levels of immune infiltration (Figure 3). In addition, tumors with low purity tend to exhibit a more active TME, which is characterized by increased expression of immune-activating or suppressive genes, as noted in our analysis (Supplementary Figure S4).

Regarding the correlation of the expression of cytokines with the level of immune infiltration, we found that the overexpression of the cytokine genes *C1QA*, *C1QB*, *C1QC*, *CXCL9*, *CXCL10*, *CXCL11* and *CXCL13* in the TME also had a negative association with the tumor purity and a positive correlation with the infiltration of the afore-mentioned immune cells in COAD (Supplementary Figure S5).

The positive correlations that we detected highlight the functional interplay between these chemokines and complement proteins with specific immune cell types in the microenvironment or colon adenocarcinomas.

Immune-related genes further regulate the recruitment, activation, and suppression of immune cells within the TIME. To determine how these genes shape the immune landscape of CRC, we also explored the correlation between their expression and the infiltration of immune cells. We found that the expression of *IL2RB*, *TIGIT*, *CCL5*, *CD96*, *HLA-DRA*, *CD8A*, *GZMH*, *FASLG*, *NKG7* and *FOXP3* exhibited a similar correlation pattern with that of immune-checkpoints and cytokines, as in the previous analysis (Supplementary Figure S6).

### 2.5. Correlation of CD8A Expression with Immune and Molecular Subtypes in CRC

**Figure 4:**
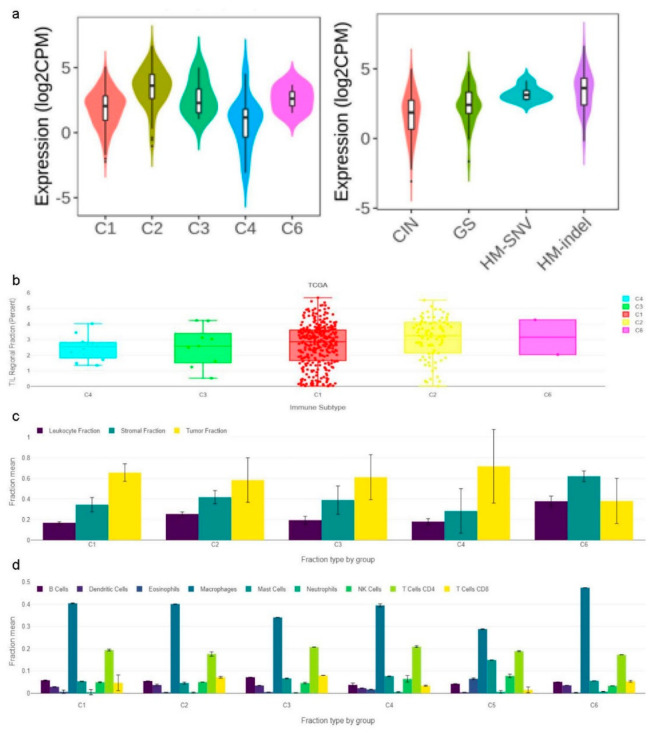
(**a**) *CD8A* expression across different immune (Pv = 4.44 × 10^−16^; C1, n = 332; C2, n = 85; C3, n = 9; C4, n = 12; C6, n = 3) and molecular subtypes (Pv = 1.87 × 10^−12^; CIN, n = 226; GS, n = 49; HM-SNV, n = 6; HM-Indel, n = 60) in COAD. (**b**) Bar plots show tumor-infiltrating lymphocyte (TIL) regional fraction stratified by immune subtypes (C1–C6) in COAD, as defined by Thorsson et al. Subtypes C1 and C2 are well represented, whereas C3–C6, particularly C6 (n = 3), contain limited numbers of samples. Accordingly, data for sparsely represented subtypes are shown for descriptive purposes only and should be interpreted with caution. (**c**) The barplots show the mean proportion of the tumor fraction, overall stromal fraction (one minus tumor fraction) and the leukocyte fraction (%) of samples with each group. Error bars show standard error of the mean. (**d**) Immune cell-type fraction (%) across C1–C6 immune subtypes in COAD.

CD8+ T cells, marked by *CD8A* expression, are critical for recognizing and destroying tumor cells via mechanisms like perforin and granzyme release. High *CD8A* expression is often associated with active anti-tumor immunity and a “hot” immune microenvironment. Therefore, we correlated *CD8A* expression with immune and molecular subtypes in COAD to find how effective cytotoxic T cell-mediated immunity is across them. As expected, we found that *CD8A* was more highly expressed in the C2 immune subtype, as well as in HM-SNV and HM-indel molecular subtypes of COAD, compared to the other immune and molecular subtypes (Figure 4a).

The higher expression of *CD8A* in the C2 immune subtype and HM-SNV/HM-indel molecular subtypes of COAD likely reflects the underlying immunological and genomic characteristics of these groups. The C2 immune subtype, often referred to as the “inflamed” or “hot” subtype, is characterized by robust immune cell infiltration and activation, including cytotoxic T cells, which explains the elevated *CD8A* expression. Similarly, HM-SNV and HM-indel subtypes, which are associated with high microsatellite instability (MSI-H), are usually TMB-high, having neoantigens that activate the immune system, and thus, leading to stronger recruitment and activation of CD8+ T cells. These findings suggest that these subtypes may benefit more from immune checkpoint inhibitors, as they harbor an immunologically active tumor microenvironment conducive to effective anti-tumor immunity.

In addition, the C2 and C6 subtypes had the highest TILs’ fraction, while C4 immune subtype tumors had the lower percentage of TILs regional fraction. On the other hand, C1 and C3 immune subtypes had similar percentage of TIL regional fraction. Given the uneven distribution of samples across immune subtypes, analyses involving C3–C6—particularly C6—were interpreted descriptively, with no statistical inference drawn from these small groups (Figure 4b). Both C2 and C6 subtypes are often characterized as “hot” immune subtypes, with high immune activity and significant infiltration of immune cells, including TILs, due to the presence of immunogenic neoantigens and active immune signaling pathways. In contrast, the C4 subtype is typically classified as “cold” or immunosuppressed, with limited immune cell infiltration and an immunosuppressive tumor microenvironment that hinders TIL recruitment. The similar TIL fractions observed in the C1 and C3 subtypes may indicate intermediate immune activity or a balance between immune exclusion and infiltration mechanisms.

We also explored the mean proportion of the tumor fraction, overall stromal fraction (one minus tumor fractions) and the leukocyte fraction of samples with each immune subgroup. In C1, C2, C3 and C4 immune subtypes, leukocyte fraction was found to have the lowest mean, while tumor fraction had the highest mean. On the contrary, leukocyte and tumor had the same fraction mean, while stromal had the highest fraction mean in the C6 immune subtype (Figure 4c). These differences reflect their distinct TME compositions across each immune subtype. In the C1, C2, C3, and C4 immune subtypes, the dominance of the tumor fraction over the leukocyte fraction suggests that they are more tumor-dense, with relatively lower immune cell infiltration, even in the immune-active C2 subtype. This could result from differences in immune cell recruitment or retention within the TME. Conversely, the C6 subtype, which is stromal-dominant, is likely characterized by extensive desmoplasia (fibrous tissue growth) and a significant stromal barrier. This may impede immune cell infiltration and tumor density, resulting in equal leukocyte and tumor fractions but a higher stromal fraction.

Finally, we examined immune cell type fraction across all immune subtypes and found that macrophages had the highest fraction mean, while neutrophils the lowest. In addition, CD4 T cells had similar fraction mean, while B cells, DCs, eosinophils, NK cells and CD8 T cells had <0.1 fraction mean. The fraction means of mast cells varied across the immune subtypes (Figure 4d). This predominance of macrophages and the low fractions of neutrophils and other immune cell types across all immune subtypes reflect the general composition of the TME in COAD. Macrophages, particularly tumor-associated macrophages (TAMs), are known to play a central role in the TME, often promoting tumor growth, angiogenesis, and immune suppression, which explains their consistently high fraction. The low fraction of neutrophils and other immune cells, suggests either a limited recruitment of these cells or their exclusion by the tumor. The variation in mast cell fractions across subtypes could indicate a subtype-specific role for these cells in modulating immune responses or promoting tumor progression.

Overall, these observations highlight the differences in the TME composition among the various immune subtypes in CRC and provide insights into the interaction between tumor cells, stromal cells, and immune cells. While C1–C4 subtypes appeared to be tumor-dominated and immune-cold, the C6 subtype was stromal-rich, with moderate immune infiltration. Understanding these differences is critical for tailoring treatments, such as targeting immune evasion in C1–C4 and stromal barriers in C6.

### 2.6. Association of Immune Infiltration with the Copy Number Aberrations Affecting Immune Checkpoints and the Most Significantly Mutated Genes in CRC

**Figure 5:**
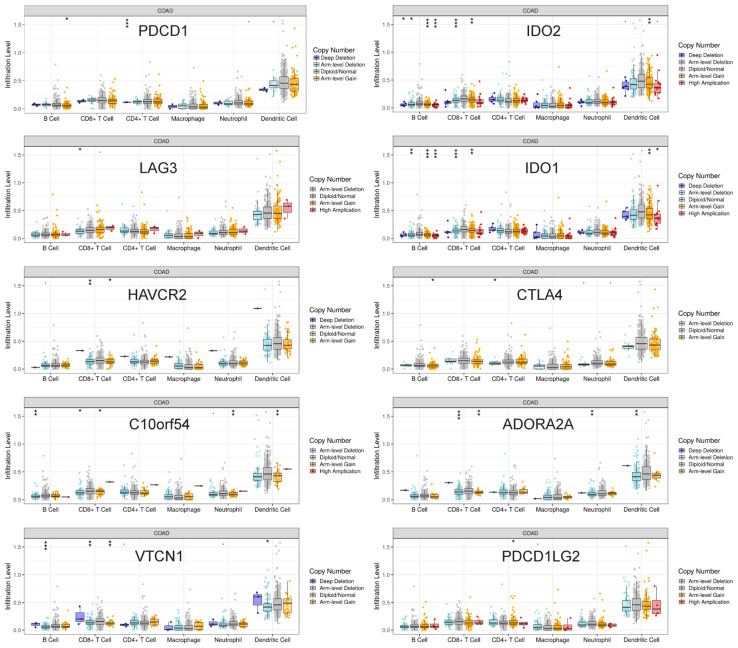
Correlation analysis between the expression of genes harboring the most frequently non-synonymous somatic point mutations or copy number aberrations with the infiltration levels of B cells, CD8^+^ T cells, CD4^+^ T cells, macrophages, neutrophils and DCs in COAD tumors. * (*p* < 0.05), ** (*p* < 0.01), and *** (*p* < 0.001).

To further investigate the relationship between genetic alterations and the TIME in CRC, we analyzed immune cell infiltration levels in COAD tumors with copy number aberrations affecting immune checkpoint loci. We observed that immune infiltration levels varied significantly depending on the copy number status of genes such as *PDCD1*, *PDCD1LG2*, *CTLA4*, *IDO1/2*, *C10orf54*, *HAVCR2*, *ADORA2A* and *VTCN1*. Tumors with diploid/normal copy numbers generally showed higher immune cell infiltration across most cell types, particularly for CD8^+^ T cells, macrophages and DCs, compared to tumors with deep deletions or high amplifications. Notably, arm-level gains also corresponded to increased infiltration for some immune cell types in specific checkpoints, such as DCs in *HAVCR2* and macrophages in *C10orf54*. However, tumors with deep deletions tended to exhibit the lowest infiltration levels, reflecting potential immune suppression associated with these aberrations (Figure 5). Furthermore, we found no significant difference in the MATH scores between the two cytolytic or immune-competent subgroups, reflecting the true differences between them (CYT-high/IC and CYT-low/ID).

Mutations in key genes can influence the infiltration of immune cells into the tumor. For example, *TP53* mutations may lead to chronic inflammation and altered immune responses [30] or *KRAS* mutations can modulate chemokine secretion, which impacts T cell recruitment [31]. Importantly, mutations in tumor cells may produce neoantigens, which can trigger immune recognition and T cell infiltration. For example, tumors with mutations in *POLE*, *MSH2*, *MSH6* (associated with MSI-H) or *BRAF* tend to show high immune infiltration and may respond well to ICIs [32,33]. Conversely, tumors with *APC* or *KRAS* mutations often exhibit immune-cold phenotypes, which require alternative or combination therapies [34]. Therefore, evaluating the infiltration in these tumors helps identify which mutations are highly immunogenic and can guide immunotherapy strategies.

To this end, we evaluated the immune infiltration levels tumors being WT or mutant for most significantly mutated genes in COAD, as previously described by the TCGA consortium [5].

**Figure 6:**
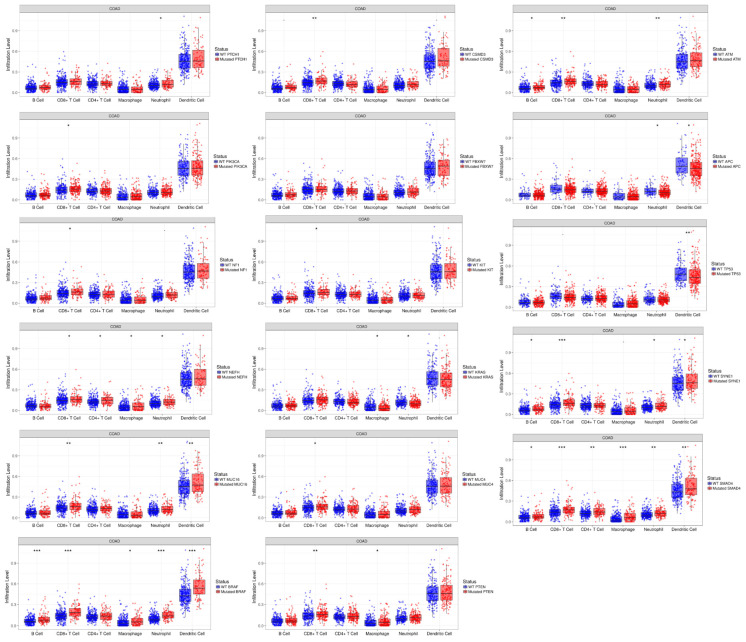
Immune cell infiltration differences between wild-type (WT) and mutant forms of the most significantly mutated genes in CRC (*ATM*, *BRAF*, *CSMD3*, *KIT*, *KRAS*, *MUC*, *MUC16*, *NEFH*, *PIK3CA*, *PTCH1*, *PTEN*, *SMAD4*, *SYNE1*, *TP53* and *APC*). Stars indicate statistically significant differences in the infiltration levels of immune cells between WT and Mut tumors (*, *p* < 0.01; **, *p* < 0.001; ***, *p* < 0.0001).

We found significantly higher infiltration of B cells in ATM^mut^, BRAF^mut^, SMAD4^mut^ and SYNE1^mut^ tumors. We also found higher infiltration of CD8^+^ T cells in ATM^mut^, BRAF^mut^, CSMD3^mut^, KIT^mut^, MUC4^mut^, MUC16^mut^, NEFH^mut^, NF1^mut^, PIK3CA^mut^, PTEN^mut^, SMAD4^mut^ and SYNE1^mut^ tumors. In addition, CD4^+^ T cells were higher infiltrated among NEFH^mut^ and SMAD4^mut^ CRCs, macrophages in BRAF^mut^, KRAS^wt^, NEFH^mut^, PTEN^mut^ and SMAD4^mut^ tumors, neutrοphils in APC^wt^, ATM^mut^, BRAF^mut^, KRAS^wt^, MUC16^mut^, NEFH^mut^, PTCH1^mut^, SMAD4^mut^ and SYNE1^mut^ tumors; and DCs in APC^wt^, BRAF^mut^, MUC16^mut^, SMAD4^mut^, SYNE1^mut^ and TP53^wt^ tumors (Figure 6).

### 2.7. Chromothripsis Across CYT-High, CYT-Low, IC and ID CRC Tumors

We have previously shown that almost one third of COADs (17/52, 32.7%) harbor chromothriptic events in their genome, the majority of which, are high confidence with or without other complex events [12]. Here, we examined more in depth the chromothriptic landscape across the various immune subgroups of CRC patients.

**Figure 7:**
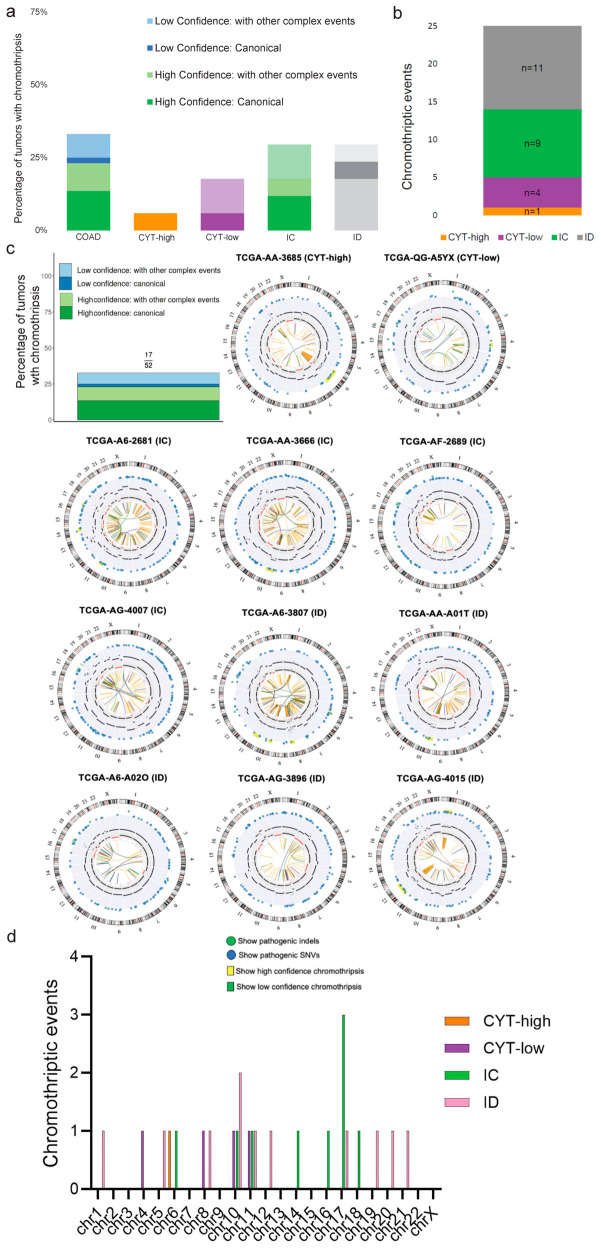
Distribution of chromothriptic events across cytolytic (CYT-high/low) and immune-competency (IC/ID) subgroups. (**a**) Percentage of CRC tumors with chromothripsis from the TCGA-COAD dataset, classified as CYT-high, CYT-low, IC and ID. Color coding of subgroup bars: orange, CYT-high; purple, CYT-low; green, immune-competent (IC); grey, immune-deficient (ID). The stacked shades indicate chromothripsis categories as defined in the legend (high-confidence canonical, high-confidence with other complex events, low-confidence canonical, and low-confidence with other complex events). (**b**) Number of chromothriptic events per cytolytic or immune competent-deficient subgroup. (**c**) Circos plots of different cytolytic and immune competency subgroups of COAD and READ tumors. (**d**) Number of chromothriptic events detected per patient in each cytolytic (CYT-high/low) or immune competency (IC/ID) subgroup. Due to the limited number of chromothriptic cases per subgroup, these data are presented descriptively and are not intended for statistical inference.

We found that 76.5% (13/17) of the patients harboring chromothriptic events were CYT-high (1/13, 5.90%), with less being CYT-low (3/13, 17.7%), IC (4/13, 23.50%) or ID (5/13, 29.4%) (Figure 7a). Notably, we identified one patient (TCGA-AA-3685) as being both CYT-high and IC. In addition, a total of 25 chromothriptic events were found across the various immune subgroups. In specific, 11 were detected in ID tumors, 9 in IC, 4 in CYT-low and one high-confidence chromothriptic event in a CYT-high patient (TCGA-AA-3685), which was associated with other complex events. In contrast, we found 4 chromothriptic events across 3 CYT-low COAD tumors: one high confidence event and another one which was linked to high confidence related with other complex events in the same patient (TCGA-QG-A5YX). We also detected 2 high confidence events associated with other complex events in 2 tumors (TCGA-A6-A56B and TCGA-A6-A567) (Figure 7b). Given the small number of chromothriptic cases in each subgroup, these observations are descriptive and do not support statistical comparisons of prevalence between groups.

It is noteworthy that CYT-high and CYT-low patients had only high confidence canonical chromothripsis (with or without other complex events), while IC and ID patients had both low and high confidence chromothripsis. Overall, we detected 9 chromothriptic events in the following five IC tumors: TCGA-AA-3686 (COAD), TCGA-A6-2681 (COAD), TCGA-AA-3666 (COAD), TCGA-AF-2689 (READ) and TCGA-AG-4007 (READ). Three low confidence events related with other complex events were found in 2 patients, 4 high confidence events in 2 patients, and 2 high confidence chromothriptic events related with other complex events, in 2 patients.

In addition, 11 chromothriptic events were identified across 5 ID tumors: TCGA-A6-3807 (COAD), TCGA-AAA02O (COAD), TCGA-AA-A01T(COAD), TCGA-AG-4015 (READ) and TCGA-AG-3896 (READ). Two high confidence events were detected in 2 tumors, 6 high confidence events related with other complex events in 3 tumors, 1 low confidence chromothriptic event in 1 tumor and 2 low confidence events associated with other complex events, were found in 2 tumors (Figure 7c). We also used circos plots to study in depth the heterogeneity of these chromothriptic events. We found that 8/13 (72.2%) of the tumors had chromothripsis in a single chromosome, while 2–4 events were recorded in the rest of the tumors. Moreover, we noted that an extensive number of chromosomes was affected by pathogenic indels and/or SNVs, as well as by both intra- and inter-chromosomal Structural Variations (SVs) (Figure 7c).

Furthermore, we explored the chromothriptic events in each cytolytic subgroup. Most of the affected chromosomes were among ID patients, while chromothripsis also significantly affected chromosomes 10 (5 events), 11 (3 events), 8 and 17 (2 events, each) among all CRC patients. In addition, 2 chromothriptic events were recorded in different chromosomes for patients TCGA-A6-2681 (IC) and TCGA-A6-3807 (ID). On the other hand, patients TCGA-AG-4007 and TCGA-AF-2689 (both IC) had events in the same chromosome (Figure 7d).

Overall, chromothriptic events were descriptively more frequently observed among CYT-low and immune-deficient tumors; however, the limited number of chromothriptic cases precludes formal statistical inference.

### 2.8. Immune-Related Subtypes Associated with Response to ICIs

The TIDE algorithm was applied to predict the responsiveness of COAD patients to ICI immunotherapy across distinct immune CYT subtypes. According to the TIDE scoring system, patients with positive scores were classified as non-responders, whereas those with negative scores were categorized as responders.

**Figure 8:**
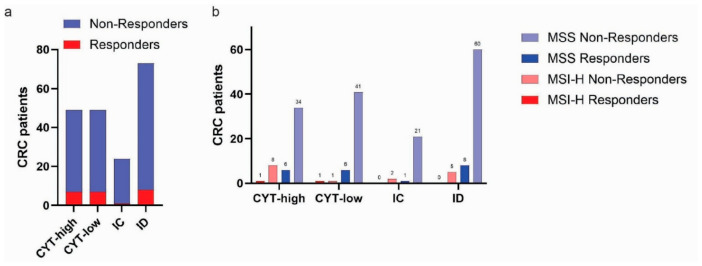
Response of CRC patients to ICI immunotherapy. (**a**) Comparison of immunotherapy outcomes among immune CYT subgroups; (**b**) evaluation of ICI response among immune CYT subgroups according to MSI-H and MSS status.

To further explore differences between CRC subgroups, we analyzed the distribution of responders and non-responders among the 195 patients included in the study. In addition to binary responder classification, the distribution of continuous TIDE scores was examined across CYT-high, CYT-low, IC, and ID subgroups. A total of 23 patients were identified as responders, comprising 7 CYT-high, 7 CYT-low, 1 IC, and 8 ID cases. Conversely, 172 patients were classified as non-responders, including 42 CYT-high, 42 CYT-low, 23 IC, and 65 ID cases. Substantial overlap in TIDE scores was observed between groups, indicating that subgroup differences are driven by score distribution rather than discrete separation. Notably, 8 non-responders exhibited both CYT-high and IC characteristics, while 15 non-responders and 2 responders displayed overlap between CYT-low and ID features (Figure 8a). These overlapping patterns highlight the substantial heterogeneity of immune activity across COAD subtypes. Negative TIDE scores in immune-deficient tumors likely reflect low modeled T-cell dysfunction due to immune exclusion rather than validated sensitivity to immune checkpoint blockade.

To evaluate how the microsatellite status influences predicted responsiveness to ICI therapy, we next examined the MSI/MSS characteristics within each group. Interestingly, 1 responder and 8 non-responders CYT-high patients, 1 responder and 1 non-responder CYT-low patient, 2 non-responders IC patients, and 5 non-responders ID patients exhibited MSI-H status (Figure 8b). This observation suggests that these individuals may still benefit from ICI immunotherapy, irrespective of their MMR/MSI classification. In contrast, 21 responders and 156 non-responders exhibited MSS status (Figure 8b), indicating that factors beyond microsatellite instability might contribute to their predicted therapeutic outcome.

Taken together, these findings suggest that while CYT-high and CYT-low COAD patients exhibit comparable prognostic outcomes COAD subtypes display substantial immunological heterogeneity and distinct immune microenvironment profiles.

To assess whether cytolytic activity has independent prognostic value, we performed multivariable Cox proportional hazards regression analysis adjusting for TNM stage and molecular subtype (MSI status). CYT status was not significantly associated with overall survival (χ^2^ = 1.44, *p* = 0.23), indicating that CYT-high and CYT-low tumors do not differ in survival outcome after accounting for established clinicopathological variables.

Similarly, TNM stage (χ^2^ = 5.69, *p* = 0.89) and MSI subtype (χ^2^ = 3.03, *p* = 0.55) were not significantly associated with survival in this cohort. The global model was not statistically significant (χ^2^ = 9.63, *p* = 0.89), suggesting limited prognostic discrimination across the variables included. Collectively, these findings indicate that CYT does not provide independent prognostic information for overall survival in TCGA-COAD.

### 2.9. GZMA and PRF1 Expression in CRC Cells Cultured Alone and in Co-Culture with T Cells

The expression of the cytotoxicity-related genes *GZMA* and *PRF1* was analyzed in HCT-116 and HT-29 CRC cell lines under monoculture conditions and following co-culture with T cells, activated using anti-CD3/CD28 dynabeads in the presence of IL-2 at an E:T ratio of 10:1. Their relative expression was quantified by qPCR, normalized to the housekeeping genes GAPDH and β-actin, and measured as fold change relative to monoculture controls.

**Figure 9:**
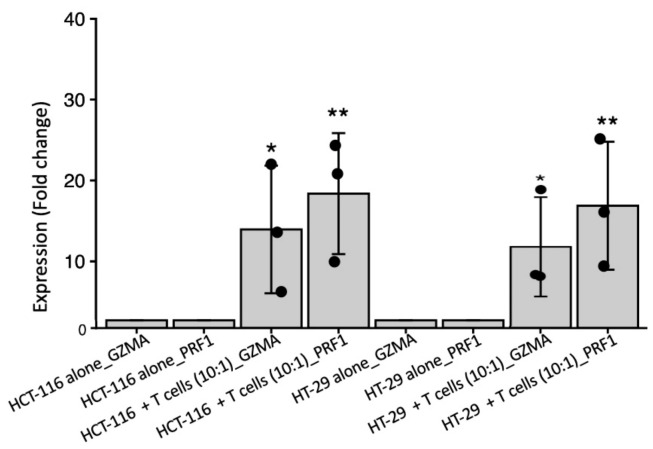
Expression of cytotoxic effector genes in CRC-T cell co-cultures. Relative mRNA expression levels of *GZMA* and *PRF1* were quantified by qPCR in CRC-T cell co-cultures and corresponding cancer cells alone controls. Each data point represents an independent biological replicate (measured in technical triplicate). Bars show mean values, and error bars indicate SD across biological replicates. * *p* < 0.05; **, *p* < 0.01 versus cancer cells cultured alone.

Following CRC–T cell co-culture, a significant increase in *GZMA* and *PRF1* transcript levels was detected in whole-well lysates compared with CRC monocultures (Figure 9). Given the mixed cellular composition of these samples and the high effector-to-target ratio, these expression changes reflect increased cytotoxic transcriptional activity within the co-culture system as a whole and cannot be attributed to a specific cellular compartment.

These results demonstrate that, in an in vitro CRC–T cell co-culture system at an E:T ratio of 10:1, T-cell exposure strongly enhances the expression of cytotoxicity-related genes within the co-cultured system. The higher induction in HCT-116 cells is consistent with the MSI-H phenotype, which is associated with increased immunogenicity and sensitivity to cytotoxic lymphocyte–mediated cues, whereas MSS HT-29 cells exhibited a comparatively lower but still robust transcriptional response. Collectively, these findings indicate enhanced cytotoxic transcriptional activity following T-cell exposure in the co-culture system.

### 2.10. LDH Cytotoxicity Assay in HCT-116 and HT-29 CRC Cell Lines

We then evaluated the cytotoxic activity of T cells against the CRC cell lines HCT-116 and HT-29 using the LDH cytotoxicity assay. Both cell lines showed a progressive reduction in cytotoxic effects with decreasing E:T ratios, demonstrating a clear E:T ratio–dependent response. At the highest E:T ratio (10:1), maximal LDH release was observed in both of them, indicating extensive tumor cell lysis.

**Figure 10:**
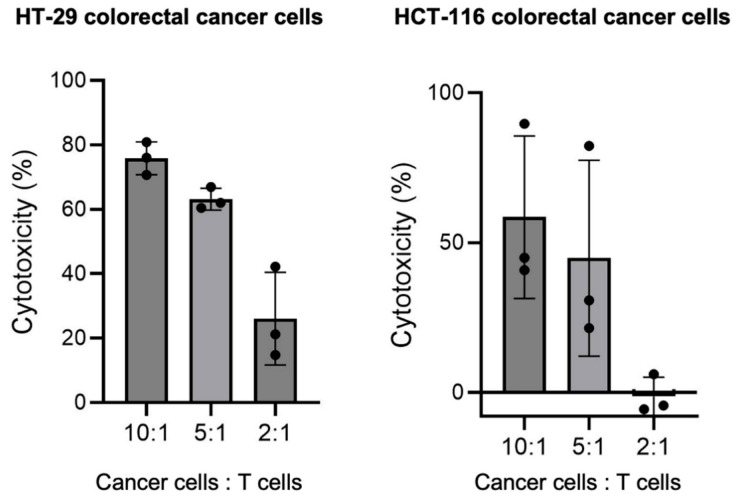
T cell-mediated cytotoxicity against HT-29 and HCT-116 cells assessed by LDH release at E:T of 10:1, 5:1, and 2:1. HT-29 and HCT-116 colorectal cancer cells were co-cultured with T cells at E:T ratios of 10:1, 5:1, and 2:1 and cytotoxicity was quantified by measuring LDH release into the culture medium. Data represents the mean ± SD. Statistical significance was determined with *p*-values indicated as *p* < 0.05.

However, HCT-116 cells consistently exhibited significantly higher LDH release compared to HT-29 across all tested E:T ratios, reflecting a greater susceptibility to T-cell–mediated cytotoxicity. In contrast, LDH release at the lowest E:T ratio (2:1) was minimal in both cell lines, indicating reduced cytotoxic activity (Figure 10). LDH release assays demonstrated effector-to-target–dependent cytotoxic activity of activated T cells against both CRC cell lines. We would like to acknowledge that given the use of polyclonally activated T cells and the absence of a non-tumor target control, the observed LDH release reflects the overall cytotoxic capacity of activated T cells in the co-culture system and may include both tumor-directed and non-specific bystander cytotoxicity.

### 2.11. CRC Cells Promote Tumor-Specific T Cell Extravasation

We next evaluated T cell extravasation and infiltration using a dynamic co-culture system in the MIVO device. This system allowed T cells to circulate beneath hydrogel-embedded CRC cells (HCT-116 and HT-29) at an E:T ratio of 10:1. The device was connected to a pumping system, generating fluid flow that circulated T cells and mimicked the circulatory dynamics of the TME.

T cell migration was assessed in response to chemoattractant-driven cues, independent of fluid flow. Positive and negative control groups were included. The positive control consisted of empty 3D alginate scaffolds without CRC cells, cultured in medium supplemented with 40% FBS as a chemoattractant. The negative control contained medium without chemoattractant and without scaffolds. Tumor samples consisted of 3D alginate cultures of HCT-116 and HT-29 cells maintained under standard conditions (MC-Coy medium with 10% FBS and 1% streptomycin–penicillin).

**Figure 11:**
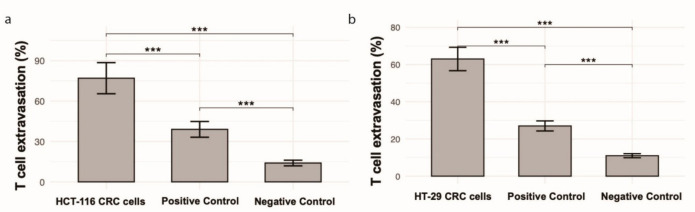
CRC cells enhance tumor-specific T cell extravasation. T cell migration toward HCT-116 (**a**) and HT-29 (**b**) alginate gels were quantified and compared to positive controls (gels without CRC cells with 40% FBS) and negative controls (RPMI with 10% FBS and 1% streptomycin–penicillin). In HCT-116, extravasation was 77%, compared to 39% in positive controls and 14% in negative controls. In HT-29, extravasation was 63%, compared to 27% in positive controls and 11% in negative controls. Data are presented as the mean ± SD (n = 6). Statistical significance: *** *p* < 0.001.

After 24 h of dynamic co-culture, migrated T cells were quantified using a hemocytometer, revealing a significant increase in immune cell migration toward the 3D tumor compartments in both CRC cell lines. In HCT-116 cultures, T cell extravasation reached 77% in the experimental group, compared to 39% in the positive control and 14% in the negative control (Figure 11a). Similarly, HT-29 cultures showed 63% migration in the experimental group, versus 27% in the positive control and 11% in the negative control (Figure 11b). This clearly shows that CRC cells actively facilitate the movement of tumor-specific T cells out of the bloodstream, exhibiting a distinct and selective pattern of immune cell infiltration within the TME.

### 2.12. Assesment of the T Cell Phenotype in Circulating, Extravasating and Infiltrating Populations

The phenotype of T cells is critical for their anti-tumor activity, as CD8^+^ T cells mediate cytotoxicity by releasing GZMA and PRF1, thereby inducing apoptosis in cancer cells. To focus on cytotoxicity, we performed a phenotypic analysis of CD4 and CD8 expressions and subsequently evaluated apoptosis in HCT-116 and HT-29 CRC cells following dynamic co-culture.

To determine whether the cytotoxicity observed at each stage was predominantly mediated by CD8^+^ T cells, we assessed if our dynamic culture system could selectively enrich specific T cell phenotypes in the extravasated and infiltrated populations. After the dynamic culture, we collected infiltrated T cells from 3D tumor hydrogels, migrated T cells from the transwell inserts, and circulating T cells. Each population was analyzed for CD4 and CD8 expression using flow cytometry. Prior to analysis, hydrogels were dissociated as previously described to generate single-cell suspensions. The results were compared across standard T cell cultures (positive control), circulating T cells from the MIVO device, migrated T cells from the transwell inserts, and infiltrated T cells from the hydrogels.

**Figure 12:**
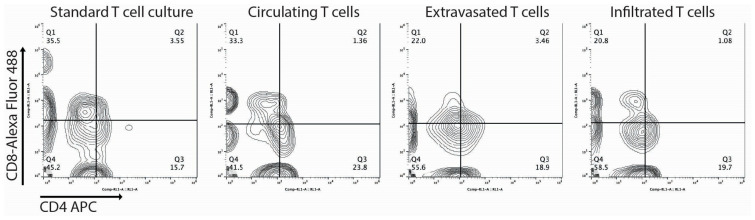
Contour plots showing the distribution of CD4^+^ and CD8^+^ T cells across different experimental compartments.

Representative flow cytometry plots show that the proportion of T cells co-expressing CD4^+^ and CD8^+^ was significantly lower in the extravasated and infiltrated groups compared with both standard T cell culture and circulating T cells, which displayed similar levels (Figure 12). The reduction in CD8^+^ T cells supports the conclusion that cytotoxicity is primarily mediated by activated CD8^+^ T cells through the expression of GZMA and PRF1. Additionally, our data suggests that CD4^+^ T cells have a relative migratory advantage or higher persistence when moving from circulation into the tissue compared to CD8^+^ T cells, which see a significant percentage reduction during the transition from the blood (circulating) to the tissue (infiltrated). The reduced CD8^+^/CD4^+^ ratio observed in the infiltrated compartment may reflect tumor-mediated TME remodeling that reduces CD8^+^ cytotoxicity. On the other hand, it could also reflect differential migratory or physical properties of T-cell subsets within the alginate matrix, rather than active tumor-mediated immune remodeling.

### 2.13. T Cell-Induced Apoptotic Mechanisms in 3D CRC Cultures

Last, we examined how T cell infiltration influences apoptotic pathways in MSI-H HCT-116 and MSS HT-29 cells using 3D CRC hydrogels. After 24 h of co-culture, tumor cells were recovered and analyzed for apoptosis and necrosis using Annexin V/PI staining, reflecting T cell–mediated cytotoxicity induced by the effector molecules GZMA and PRF1.

**Figure 13:**
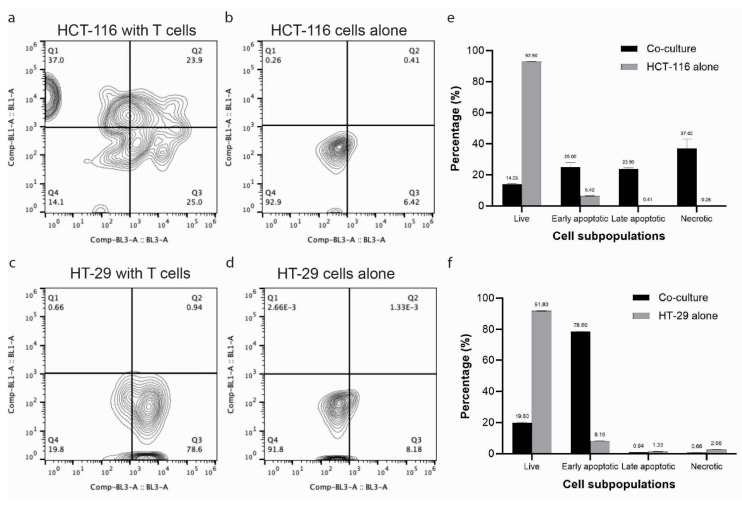
T cell–mediated apoptosis and necrosis in 3D CRC cultures. (**a**–**d**) Representative flow cytometry plots showing viable (Annexin V^−^/PI^−^), early apoptotic (Annexin V^+^/PI^−^), late apoptotic (Annexin V^+^/PI^+^), and necrotic (Annexin V^−^/PI^+^) populations. (**e**,**f**) Quantification of cell death subpopulations demonstrates that HCT-116 cells undergo mixed apoptosis–necrosis (necrosis 37.0%, early apoptosis 25.0%, late apoptosis 23.9%) (n = 9), whereas HT-29 cells predominantly display early apoptosis (78.6%), with limited late apoptosis (1.3%) and necrosis (2.7%). Control: HCT-116 and HT-29 3D monoculture.

In HCT-116 cells, T cell infiltration reduced viability to 14.1%, with necrosis predominating (37.0%) alongside early (25.0%) and late apoptosis (23.9%), indicating that immune-mediated killing engages multiple cell death pathways. HCT-116 monoculture remained highly viable (92.9%), confirming that T cell contact is required to trigger apoptosis (Figure 13a–c).

HT-29 cells displayed a distinct pattern: viability decreased to 19.8%, driven primarily by early apoptosis (78.6%), with limited late apoptosis (1.3%) or necrosis (2.7%). Monocultures retained >91% viability. These results suggest that in MSS CRC, T cells initiate apoptotic signaling but do not efficiently drive terminal cell death within the same timeframe (Figure 13d–f).

Overall, these findings reveal subtype-specific apoptotic mechanisms: MSI-H HCT-116 cells are highly susceptible to T cell–mediated mixed apoptosis-necrosis, HT-29 cells displayed a distinct apoptotic profile after 24 h of co-culture. Although overall viability was reduced, cell death was characterized predominantly by early apoptosis (Annexin V^+^/PI^−^, 78.6%), with minimal late apoptosis or necrosis. This pattern suggests that, at the assessed time point, MSS HT-29 cells initiate apoptotic signaling but progress more slowly toward terminal cell death compared with MSI-H HCT-116 cells.

The 3D model effectively recapitulates differential T cell–tumor interactions and apoptotic dynamics, providing a physiologically relevant platform to study CRC immunogenicity.

## 3. Discussion

CRC remains a leading cause of cancer morbidity and mortality globally [1,35], with outcomes heavily influences by the TME and immune contexture. Importantly, CYT and CD8^+^ T cell abundance represent related but non-redundant immune features. CYT reflects cytotoxic functional activity, whereas CD8A/CD8B-based metrics quantify immune cell infiltration. Understanding how immune cell composition and function shape tumor behavior and therapeutic response is essential for improving precision immunotherapy strategies [8,9,36,37,38]. In this study, we characterized the TME across different cytolytic or immune-competent/deficient CRC subgroups to better understand the mechanisms underlying differential responses to ICI immunotherapy.

Immune cells are central to the TME, influencing cancer progression and therapy [19,20]. Our integrative analyses revealed that IC and CYT-high CRC tumors are enriched in effector populations such as CD8^+^ T cells, activated CD4^+^ T cells, M1 macrophages, and B cells, with concomitantly higher stromal and leukocyte fractions, consistent with enhanced immune surveillance. In contrast, ID and CYT-low tumors were characterized by increased M2 macrophages, CD4^+^ T cells, NK cells, and neutrophils, indicative of a suppressive or poorly coordinated TME. These findings align with established evidence that effector T cells and pro-inflammatory macrophages correlate with favorable anti-tumor immunity, whereas M2 polarization and regulatory phenotypes support tumor progression and immune evasion [24,39,40,41,42,43,44,45,46]. The immune landscape defined by CYT and IC status reflects a fundamental dichotomy between “hot” and “cold” tumors: CYT-high/IC tumors exhibit coordinated effector infiltration and immune activation, while CYT-low/ID tumors exhibit features of immune suppression. Prior work across cancer types has similarly stratified tumors based on immune CYT and linked these profiles to prognosis and immunotherapy response [17,25,47,48].

The classification of tumors into immune-competent and immune-deficient groups is based on a binary cutoff applied to the continuous ESTIMATE score. As a result, tumors with ESTIMATE values near zero may not differ biologically in a strictly dichotomous manner. While this approach provides an intuitive and biologically grounded stratification of immune-enriched versus immune-depleted tumors, it does not fully capture the continuum of immune variation within the tumor microenvironment. Importantly, the key associations observed in this study are driven primarily by CYT, analyzed as a continuous variable independent of ESTIMATE score, thereby reducing reliance on the IC/ID grouping.

Immune cell composition has been repeatedly linked to clinical outcomes in CRC. High CD8^+^ T cell infiltration is associated with improved survival, whereas increased presence of immunosuppressive populations, including M2 macrophages and regulatory T cells, often predicts poor prognosis [44,45,46,49]. Mast cells, NK cells, and macrophage subtypes have also been implicated in modulating patient outcomes, underscoring the complexity of immune regulation in CRC [44]. Our findings align with these observations and further demonstrate that immune infiltration patterns are not uniform but instead reflect distinct immune states shaped by CYT status and immune subtype classification.

To investigate how immune activation and immune evasion coexist within the CRC TME, we analyzed correlations between immune cell infiltration and immune checkpoint expression. We observed that immune checkpoints such as PDCD1, CD274, CTLA-4, LAG3, IDO1, HAVCR2, TIGIT, and TNFRSF9 exhibit context-dependent associations with immune infiltration. In some CRCs, checkpoint overexpression coincided with relatively low immune infiltration but enrichment of specific immune subsets, including CD8^+^ T cells, monocytes, eosinophils, and dendritic cells, suggesting early or spatially restricted immune activation [50]. In contrast, particularly in MSI-H/dMMR tumors, high checkpoint expression correlated with extensive immune infiltration, low tumor purity, and poor survival, reflecting chronic antigen exposure, immune exhaustion, and adaptive immune resistance [51]. The prognostic value of PD-1–expressing tumor-infiltrating lymphocytes, especially in pMMR CRC, further emphasizes the dual role of checkpoint pathways in immune activation and dysfunction [52].

While CYT-high tumors exhibited increased immune infiltration and cytotoxic gene expression, this immune activation was not associated with reduced metastatic incidence or improved survival. The comparable stage IV distribution between CYT subgroups suggests that cytolytic activity alone is insufficient to constrain disease progression in colorectal cancer. Accordingly, CYT should not be interpreted as a prognostic or predictive biomarker, but rather as a descriptor of immune contexture.

Additionally, the qPCR analysis of *GZMA* and *PRF1* was performed on mixed co-culture lysates containing both CRC cells and activated T cells. As such, the observed increase in these transcripts cannot be interpreted as CRC cell-intrinsic transcriptional responses but rather reflects enhanced cytotoxic gene expression within the co-culture system. Definitive attribution of *GZMA* and *PRF1* expression to tumor or immune compartments would require cell-fractionation-based RNA isolation or single-cell approaches, which were beyond the scope of the present study.

Although no global survival difference was observed between CYT-high and CYT-low COAD patients, the identified CRC subtypes exhibited distinct immunological and molecular characteristics, highlighting the substantial heterogeneity of the tumor immune microenvironment across COAD. MSI-H/dMMR tumors exemplify this paradigm, as they are characterized by high TMB, dense CD8^+^ T-cell infiltration, and favorable responses to ICI therapy [37,38,53]. However, even within MSI-H CRC, acquired resistance frequently emerges following initial responses, driven by T-cell exhaustion, upregulation of alternative immune checkpoints, and evolving immune escape mechanisms [54]. Importantly, it is worth mentioning that TIDE was not trained or clinically validated in MSS colorectal cancers, and therefore, predicted responsiveness in immune-excluded tumors should not be interpreted as evidence of clinical benefit.

To complement our bioinformatic findings, we conducted functional in vitro experiments to directly assess CD8^+^ T-cell cytotoxicity against CRC cells. Activated CD8^+^ T cells demonstrated robust, dose-dependent cytotoxicity against both HCT-116 (dMMR/MSI-H) and HT-29 (MSS) cell lines, as measured by LDH release assays. Notably, HCT-116 cells exhibited significantly greater sensitivity to T cell-mediated killing, underscoring the heightened immunogenicity of MSI-H tumors. These results are consistent with previous studies showing enhanced T cell cytotoxicity against CRC cells at increasing effector-to-target ratios and reinforce the critical role of effector cell dose in maximizing antitumor activity [33,55,56].

A limitation of the LDH cytotoxicity assays is the absence of a non-tumorigenic or irrelevant target cell line control. Polyclonal activation of T cells with anti-CD3/CD28 Dynabeads and IL-2 can induce non-specific cytotoxicity mediated by soluble factors such as IFN-γ and TNF-α. As a result, the LDH release observed in CRC co-cultures may reflect a combination of tumor-directed and bystander cytotoxic effects rather than strictly tumor-antigen-specific killing. Future studies incorporating appropriate non-tumor targets will be required to distinguish these components.

At the transcriptional level, co-culture with CRC cells induced upregulation of key cytotoxic effector genes, including *PRF1* and *GZMA*, in activated T cells. Although the magnitude of induction was moderate compared with polyclonal stimulation using CD3/CD28 and IL-2, these findings indicate that direct tumor contact initiates cytotoxic programs. Prior studies have demonstrated that maximal effector differentiation requires cytokine support, particularly IL-2, and that tumor-derived immunosuppressive signals can dampen perforin and granzyme expression [26,27,57]. Nevertheless, the co-expression of *PRF1* and *GZMA* remains a validated surrogate marker of immune CYT across multiple cancer types, including CRC [27,47]. Importantly, GZMA-expressing CD8^+^ T cells have been shown to directly induce tumor cell death in CRC, supporting the functional relevance of our transcriptional observations [58].

Using a dynamic 3D co-culture system, we further demonstrated that CRC cells actively promote tumor-specific T-cell extravasation and infiltration. Both HCT-116 and HT-29 cultures enhanced T-cell migration compared with chemoattractant-only controls, indicating that tumor-derived signals facilitate immune cell recruitment. The stronger migration toward HCT-116 cells aligns with the increased immunogenicity of MSI-H tumors. Despite comparable infiltration, downstream cytotoxic outcomes were subtype specific. In HCT-116 cultures, T cells induced extensive tumor cell death through combined apoptotic and necrotic mechanisms, consistent with effective perforin–granzyme-mediated killing. In contrast, HT-29 cells predominantly underwent early apoptosis with minimal late apoptosis or necrosis, suggesting intrinsic resistance to terminal T-cell–mediated cytotoxicity. These findings mirror clinical observations that MSS CRC often exhibits limited responsiveness to immunotherapy despite immune cell infiltration.

The predominance of early apoptotic HT-29 cells following 24 h of co-culture should be interpreted with caution. Annexin V^+^/PI^−^ staining reflects early apoptotic events and does not, by itself, indicate irreversible cell death or immune resistance. It is possible that MSS CRC cells progress more slowly through the apoptotic cascade, such that at later time points they may undergo late apoptosis or secondary necrosis. A formal time-course analysis extending beyond 24 h would be required to distinguish delayed apoptosis from genuine resistance or apoptotic recovery, and this represents a limitation of the current study.

The divergent apoptotic kinetics between HCT-116 and HT-29 cells are consistent with prior reports indicating enhanced susceptibility of MSI-H CRC cells to cytotoxic lymphocyte–mediated killing relative to MSS tumors [59,60].

Beyond immune composition, genomic alterations significantly shaped the tumor immune microenvironment. Tumors with diploid copy number profiles exhibited higher immune infiltration, whereas deep deletions were associated with markedly reduced immune presence, indicating that genomic stability in immune-related loci supports active immune surveillance. Arm-level gains were associated with increased infiltration of specific immune populations, such as macrophages and dendritic cells, highlighting the complex effects of copy number alterations on immune dynamics [3,15,61]. Furthermore, mutations in key CRC driver genes, including ATM, BRAF, PTEN, and SMAD4, were associated with distinct immune infiltration patterns. ATM- and BRAF-mutant tumors displayed elevated infiltration of CD8^+^ T cells and dendritic cells, suggesting an inflamed and potentially immunogenic TME, whereas macrophage and neutrophil enrichment in BRAF-mutant, KRAS wild-type, and MUC16-mutant tumors may reflect immunosuppressive or pro-tumorigenic immune states depending on polarization.

We also investigated chromothripsis, a catastrophic mutational process frequently observed in CRC and associated with poor prognosis [12,62]. Chromothriptic events were more prevalent in CYT-low and ID tumors and often involved extensive intra- and inter-chromosomal rearrangements affecting chromosomes 8, 10, 11, and 17. The analysis of chromothripsis in relation to immune cytolytic and immune-competency subgroups is limited by the small number of cases harboring chromothriptic events. As a result, no statistically powered comparisons could be performed, and the observed distributions should be interpreted descriptively. Larger cohorts with comprehensive whole-genome sequencing will be required to determine whether chromothripsis is differentially associated with immune cytolytic activity or immune deficiency in colorectal cancer.

Chemokine and complement signaling further contributed to shaping the immune landscape. CXCL9, CXCL10, and CXCL11, which recruit effector T cells and NK cells via CXCR3, were associated with increased immune infiltration and improved immune-mediated tumor control, consistent with prior CRC studies [63,64]. CXCL13 expression, linked to B-cell recruitment and tertiary lymphoid structure formation, may further enhance local antitumor immunity. In contrast, C1QA, C1QB, and C1QC expression correlated with macrophage infiltration and may influence macrophage polarization and immune suppression, suggesting potential roles for complement-targeted therapeutic strategies in specific CRC subsets.

Collectively, our findings demonstrate that effective antitumor immunity in CRC requires both efficient T-cell infiltration and sustained cytotoxic activity, which are shaped by immune contexture, checkpoint signaling, chemokine gradients, and genomic alterations. CYT-high and immune-competent tumors emerge as strong candidates for immunotherapy, whereas MSS and CYT-low tumors present substantial challenges due to immune exclusion, intrinsic resistance, and immunosuppressive TME features. Our data suggest that combining ICIs with strategies that enhance T-cell recruitment, boost effector function, and reprogram suppressive components of the TME may be essential to improve outcomes, particularly in immunologically “cold” MSS CRC.

In conclusion, this study provides a comprehensive immunogenomic and functional framework for understanding immune heterogeneity in CRC. Integration of CYT scores, immune subtypes, chemokine and checkpoint profiles, and genomic features may enable improved patient stratification and guide the development of precision immuno-oncology strategies. Future studies should focus on biomarker-driven combination therapies aimed at overcoming immune resistance and expanding the benefits of immunotherapy to a broader population of CRC patients.

## 4. Materials and Methods

### 4.1. Data Collection

We acquired RNA-sequencing data (htseq counts) from 458 colon adenocarcinomas (COAD) and 167 rectum adenocarcinomas (READ) from the TCGA database (https://portal.gdc.cancer.gov/). All RNA-seq and clinical data stemmed from tumor tissues and their adjacent normal tissues. All tumor samples were untreated primary colorectal cancers and analyzed, as previously described [17]. Analyses were primarily performed using the TCGA-COAD cohort due to its larger sample size, while TCGA-READ samples were included where available but were not analyzed as an independent cohort because of limited statistical power. Ethics approval and informed consent were not required in this study since the data were obtained from publicly available databases. PBMCs were obtained from anonymized healthy donors under informed consent for research use, according to institutional and national regulations in Cyprus.

### 4.2. Identification of Immune Subtypes and Calculation of CYT

CRC has a highly heterogeneous tumor immune microenvironment (TIME), which varies from “immune-inflamed” to “immune-desert” or “immune-excluded” phenotypes. We calculated the Differentially Expressed Genes (DEGs) using the “*limma*” package (Bioconductor, Release 3.23) and voom transformation. The |log(FC)| > 1 and False Discovery Rate (FDR) *p*-value < 0.1 were set as the cutoffs to screen DEGs, as previously described [65,66]. The DEGs were compared to a total of 2498 immunologically relevant genes, retrieved from the Immunology Database and Analysis Portal (ImmPort) (https://www.immport.org/) database [67].

We then stratified tumors into immune subtypes, using unsupervised complete linkage clustering. The 1046 most variable immune-related genes were used to infer the immune subtype classification of CRCs. Differences in the immune gene expression between tumors and control samples, were assessed using Wilcoxon’s test.

The immune subtype of each colon adenocarcinoma sample was defined by comparing its TME characteristics, based on the predicted presence of infiltrating stromal and immune cells in each tumor tissue. The difference of the above-mentioned scores, termed as ‘ESTIMATE score’ was used to infer tumor purity and define each immune subtype using ESTIMATE [68]. Finally, to confirm the significance of the TME characteristics in the immune-competent (IC) tumors, we compared the CYT index with the ‘immune’, ‘stromal’ and ‘ESTIMATE’ scores. The zero threshold for ESTIMATE score was used in accordance with the original ESTIMATE framework, where positive values indicate immune- and stromal-enriched tumors, while negative values indicate immune-sparse tumors. We acknowledge that ESTIMATE score is a continuous variable and that samples near the zero boundary may exhibit intermediate immune characteristics. The immune-competent and immune-deficient classification was based on a binary cutoff applied to a continuous ESTIMATE score, which may oversimplify immune heterogeneity in tumors near the classification boundary.

Gene expression data were retrieved from three different platforms, Agilent G4502A (218 samples), RNA-Seq (81 samples), and RNA-Seq-V2 (263 samples). Samples with a negative ESTIMATE score were classified as immune-deficient (ID) and those with a positive ESTIMATE score, as immune-competent (IC). Based on this criterion, our cohort was composed of 255 immune deficient and 307 immune competent tumors, respectively (Supplementary Table S1). Tumor Immune Estimation Resource (TIMER, http://cistrome.org/TIMER; accessed on 10 January 2025) was used to analyze the relationship between immune checkpoints’, cytokines’ and other immune-related genes’ expression and immune infiltration levels (B cells, CD4^+^/CD8^+^ T cells, neutrophils, macrophages and DCs) in the TME of CRCs [52,69]. Results with criteria (*p* < 0.05 and |Pearson’s rho| > 0.2) were considered to have significant correlation between them. For all correlation analyses involving immune gene signatures, cytokines, and immune cell infiltration, *p*-values were adjusted for multiple testing using the Benjamini–Hochberg false discovery rate (FDR) procedure. Only correlations with FDR-adjusted *p* < 0.05 were considered statistically significant. CIBERSORT (https://cibersortx.stanford.edu/), was used to compute the proportion of 22 distinct immune cell infiltrates.

The cytolytic index (CYT) in each CRC sample was calculated using the geometric mean of the expression of *GZMA* and *PRF1*, as previously explained [47], to capture cytotoxic effector activity rather than immune cell abundance. This definition intentionally excludes lineage markers such as *CD8A* and *CD8B*, which reflect T-cell quantity, and focuses instead on genes encoding the core granzyme–perforin cytolytic machinery. Patients were then classified into a CYT-high and a CYT-low cytolytic subgroups, based on the upper or lower 25th quartiles of the cytolytic index, respectively [17].

Multivariable Cox proportional hazards regression analysis was performed to assess the prognostic value of cytolytic activity (CYT), adjusting for TNM stage and MSI status. Hazard ratios (HRs) and 95% confidence intervals (CIs) were calculated using the survival package in R (v3.8-6).

### 4.3. Consensus Molecular Subtype (CMS) Classification

RNA-seq gene expression data for TCGA colon adenocarcinoma (TCGA-COAD) were obtained using the TCGAbiolinks R package (v2.40.0) from the Genomic Data Commons, using the “STAR—Counts” workflow. Raw counts were log2-transformed after filtering genes with missing or empty annotations and mapping Ensembl identifiers to gene symbols based on GDC-provided annotations. CMS classification was performed using a signature-based approach, whereby predefined gene sets representing CMS1–CMS4 were scored in each sample by calculating the mean expression of genes within each signature, and samples were assigned to the subtype with the highest score [2]. All analyses were conducted in R (v4.6).

### 4.4. Functional Enrichment Analysis of DEGs

We determined the gene sets and pathways in which immune cytolytic subgroups of tumors were enriched using Kyoto Encyclopedia of Genes and Genomes (https://www.genome.jp/kegg/; accessed on 20 January 2025) and Gene Ontology (https://geneontology.org/; accessed on 20 January 2025) functional enrichment analysis.

### 4.5. Immune Signatures and Cell Fractions

We extracted immune signatures and cell type fractions in each immune cytolytic subgroup from the Cancer Immunome Atlas (TCIA) (https://tcia.at/; accessed on 10 February 2025) [70]. Immune signatures were expressed in log_2_(TPM + 1) and presented as heatmaps of the average expression values. In specific, we extracted 35 gene signatures specific for neutrophils, NK cells, monocytes, MDSCs, macrophages, immature DCs, activated DCs, eosinophils, CD56^bright^ DCs, NK T-cells, plasmacytoid DCs, mast cells, CD56^Dim^ NK cells, activated CD4 cells, immature B cells, type 2 helper T cells, activated B cells, effector memory CD4 T cells, activated CD8 T cells, central memory CD4 T cells, central memory CD8 T cells, type 17 helper cells, effector memory CD8 cells, Tregs, memory B cells, type 1 helper cells, gamma-delta T cells, T follicular helper cells, MHC-classical class-I, MHC-classical class-II, MHC-non-class class-I, MHC-non-class class-II, cancer germline antigens, immunostimulators and immunoinhibitors (Supplementary Table S2).

### 4.6. Correlation of CYT with the Expression of Immune Checkpoints, Cytokines, Other Immune-Related Genes and the Level of Immune Infiltration

We then correlated the expression of immune checkpoints with CYT across all tumor samples, using TIMER 2.0. (http://timer.cistrome.org/; accessed on 20 February 2025). The expression (log_2_RSEM) of 11 immune checkpoints (*PDCD1 (PD-1)*, *CD274 (PD-L1)*, *PDCD1LG2 (PDL2)*, *CTLA-4*, *IDO1*, *IDO2*, *HAVCR2 (TIM3)*, *ADORA2A (A2AR)*, *LAG3*, *VISTA (C10orf54)* and *VTCN1 (B7-H4)*) was also correlated with the immune infiltration level in each tumor sample, using the Spearman’s rank test, after correcting for the tumor’s purity [68].

CYT was also correlated with the expression of cytokines (*C1QA*, *C1QB*, *C1QC*, *CXCL9*, *CXCL10*, *CXCL11* and *CXCL13*) and other immune-related genes *(IL2RB*, *TIGIT*, *CCL5*, *CD96*, *HLA-DRA*, *CD8A*, *GZMH*, *FASLG*, *NKG7* and *FOXP3*), as previously described [17].

We further explored *CD8A* expression across the different immune subtypes (C1, C2, C3, C4 and C6) as termed by Thorsson et al. (2018) [48], as well as across tumors with chromosomal instability (CIN), genomically stable (GS), hypermutated-insertion deletion mutation (HM-indel), and hypermutated-single-nucleotide variant predominant (HM-SNV) tumors [71].

### 4.7. Distribution of Immune Infiltration Under Different Gene Mutations

We investigated whether genes harboring the most frequently non-synonymous somatic point mutations (top 10%) or somatic copy number aberrations (SCNA) [17] associate with the abundance of immune infiltrates. The statistical significance of the distribution of immune infiltration levels under different gene mutation status in each immune subset, was estimated using two-sided Wilcoxon rank sum test.

SCNAs included deep deletion (−2), arm-level deletion (−1), diploid/normal (0), arm-level gain (1) and high amplification (2), as defined by GISTIC 2.0 [61]. We explored the distribution of each immune subset at each copy number status and compared the infiltration level for each SCNA in CRC with the normal using two-sided Wilcoxon rank sum test.

CRCs with no available genomic instability data were excluded from the analysis. Silent and non-silent mutation rates were the number of mutations divided by target length of the genome in Mb. In the SCNA burden, the ‘number of segments’ was equal to the total number of segments in each sample’s copy number profiles.

### 4.8. Chromothripsis

We further explored the rates of chromothripsis (i.e., shattering of one or more chromosomes into numerous fragments, which are then reassembled in a disorganized manner) with low and high confidence (canonical or with other complex events) across the different cytolytic subgroups (CYT-high, CYT-low), as well as across the different ‘immune-competency’ subgroups (IC and ID) of CRCs. For the analysis we used data extracted from the PCAWG cohort, using Chromothripsis explorer (http://compbio.med.harvard.edu/chromothripsis/; accessed on 20 March 2025) with the following criteria: 6 breakpoints in chromothripsis region, 4 CN oscillations, 4 CN oscillations across 3 CN states, 0.13 purity and 1.283 ploidy. Circos plots were used to visualize complex mutational profiles, as previously described [12].

### 4.9. Intra-Tumoral Heterogeneity

Intra-tumoral heterogeneity was measured, by calculating the width of the Variant Allele Frequencies (VAF) distribution, and a Mutant-Allele Tumor Heterogeneity (MATH) score was assigned to each sample as the ratio of the width to the center of its distribution of mutant-allele fractions among tumor-specific mutated loci [17].

### 4.10. Copy Number Aberrations and Cancer Driver Genes

The identification of genomic regions with significant amplifications or deletions (CNAs) and of cancer driver genes, based on positional clustering, was performed using GISTIC v2 analysis and the MAF files [72]. A G-score considering the amplitude of the aberration, as well as the frequency of its occurrence across each tumor sample was assigned. We calculated the CNAs by keeping the sum of segment mean changes ≥ 0.6 and ≤−0.4 between somatic and normal samples. Regions with FDR q-values  <  0.1 were considered significant.

### 4.11. Prediction of Cancer Neoepitopes

We estimated the cellular composition of cancer neoantigens per Mb across CYT and IC/ID subgroups, using data from TCIA (https://tcia.at/; accessed on 1 April 2025) [73,74].

### 4.12. Prediction of Response to ICI Therapy

Tumor Immune Dysfunction and Exclusion (TIDE) (http://tide.dfci.harvard.edu/; accessed on 15 April 2025) was used to predict patient responses ICI therapy within each immune CYT subgroup. TIDE assesses tumor immune evasion from pre-treatment gene expression by modeling two key mechanisms: T-cell dysfunction in tumors with high T-cell infiltration and T-cell exclusion in tumors with low infiltration. T-cell abundance was estimated using the expression of *CD8A*, *CD8B*, *GZMA*, *GZMB*, and *PRF1*. Dysfunction and exclusion scores were calculated based on predefined gene signatures and integrated into a composite TIDE score, with higher scores reflecting greater immune evasion. Patients were subsequently categorized as responders (low TIDE scores) or non-responders (high TIDE scores) to ICI therapy [75].

For the estimation of CD8^+^ T-cell abundance within the TIDE framework, a broader gene set (CD8A, CD8B, GZMA, GZMB, PRF1) was used to capture both lineage identity and cytotoxic potential of CD8^+^ T cells.

### 4.13. CRC Cell Lines and T-Cell Preparation

HCT-116 and HT-29 colorectal cancer cell lines were cultured in McCoy’s 5A medium supplemented with 10% fetal bovine serum (FBS) and 1% penicillin–streptomycin and maintained at 37 °C in a humidified atmosphere containing 5% CO_2_.

T cells were generated from human peripheral blood mononuclear cells (PBMCs) isolated from healthy donors by Ficoll density gradient centrifugation. PBMCs were activated using anti-CD3/CD28 Dynabeads (Thermo Fisher Scientific, Monza, Italy), as per the manufacturer’s instructions, in the presence of 30U/mL recombinant human interleukin-2 (IL-2) (Thermo Fisher Scientific, Monza, Italy). Activated T cells were cultured and expanded in RPMI-1640 medium supplemented with 10% FBS and 1% penicillin–streptomycin at 37 °C in a humidified atmosphere containing 5% CO_2_ for 24 or 48 h prior to co-culture and cytotoxicity assays and used throughout all assays.

### 4.14. Three-Dimensional (3D) CRC Cell Culture

3D tumor models were generated using HCT-116 and HT-29 CRC cells, as previously described [57,76]. Briefly, both CRC cell lines were resuspended in a 1:1 (*v*/*v*) mixture of McCoy’s medium (supplemented with 10% FBS and 1% penicillin/streptomycin) and a 1% alginate solution, resulting in a final alginate concentration of 0.5% (*w*/*v*).

The resulting cell suspension was dropped into a 0.5 M CaCl_2_ gelling bath to allow spheroid formation. Subsequently, the hydrogels were washed with distilled water and transferred to a 96-well plate. The 3D cultures were maintained overnight in McCoy’s medium supplemented with 10% FBS, 1% penicillin/streptomycin, and 5 mM CaCl_2_ to ensure hydrogel stability.

### 4.15. Dynamic Cell Culture of HCT-116 and HT-29 CRC Cells with T Cells

Dynamic co-cultures were established using the Single-Flow MIVO Device (React4Life, Genoa, Italy) to recapitulate a 3D dynamic TME. T cells were counted and resuspended in RPMI-1640 medium supplemented with 10% FBS, 1% penicillin/streptomycin, and 30 IU/mL IL-2 (per 1 × 10^6^ cells). The cell suspension was loaded into the MIVO chamber (1.5 mL volume) to achieve an E:T ratio of 10:1 with HCT-116 and HT-29 cells.

T-cell circulation was generated using a pumping system set at a flow rate, according to the manufacturer’s instructions, to simulate capillary-like flow conditions. Three-dimensional HCT-116 and HT-29 alginate hydrogels were cultured in a separate compartment and physically isolated from the circulating T cells by a permeable porous membrane, allowing the diffusion of soluble factors without direct cell–cell contact.

Positive and negative controls for cell migration were included. The positive control consisted of empty 3D alginate scaffolds (without tumor cells) cultured in RPMI-1640 supplemented with 40% FBS and 1% penicillin/streptomycin to act as a chemoattractant. The negative control consisted of conditions lacking alginate scaffolds and chemoattractant supplementation, using RPMI-1640 (Invitrogen, Toulouse, France) containing 10% FBS only, to assess background migration.

### 4.16. Extravasation and Infiltration of T Cells in HCT-116 and HT-29 3D Tumor Models

To assess T-cell extravasation and infiltration, cells were collected after 24 h of dynamic co-culture from the medium surrounding the 3D tumor hydrogels within the transwell inserts. This collection enabled the quantification of the proportion of T cells that extravasated from the capillary-like circuit, serving as a model of the human bloodstream.

T cells were also collected from the circulating compartment to quantify the proportion of cells remaining in circulation across experimental conditions, including positive and negative controls. Both circulating (capillary circuit) and extravasated (tumor niche) T cells from triplicate samples and control groups were harvested and counted using a hemocytometer Bioanalytic GmbH (Freiburg, Germany). These measurements were used to calculate the proportions of T cells undergoing extravasation and tumor infiltration, as well as their subsequent contribution to tumor cell apoptosis.

### 4.17. Flow Cytometry Analysis

Flow cytometry was performed to characterize the phenotype of standard, circulating, extravasating, and infiltrating T-cell populations. Circulating T cells were recovered from the MIVO device, migrated T cells from the transwell inserts, and infiltrating T cells from the 3D hydrogels. These populations were compared with standard T-cell cultures used as positive controls. Cells were washed with Ca^2+^/Mg^2+^-free PBS and incubated for 30 min at room temperature in the dark with fluorochrome-conjugated antibodies against CD4 and CD8. T cells were stained using anti-human CD4-APC and anti-human CD8 (green fluorophore) antibodies.

Apoptosis of HCT-116 and HT-29 cells following dynamic co-culture was also evaluated by flow cytometry. Cancer cells were recovered from 3D alginate scaffolds by dissolving the alginate matrix using an alginate-solubilizing solution (0.15 M NaCl, 100 mM trisodium citrate dihydrate) and incubating for 30 min at 37 °C in a humidified atmosphere containing 5% CO_2_, according to the manufacturer’s instructions.

Recovered cells were washed with Ca^2+^/Mg^2+^-free PBS and stained with Annexin V and PI to discriminate live, early apoptotic, late apoptotic, and necrotic cell populations. Briefly, cells were washed, centrifuged, resuspended in ice-cold PBS, and centrifuged again. Supernatants were discarded, and cell pellets were stained with FITC–Annexin V and PI solution. Samples were incubated for 15 min at room temperature in the dark. In parallel, apoptosis of immune cells isolated from all experimental stages was evaluated to confirm their viability and to ensure that the apoptosis detected in cancer cells resulted from immune cell-mediated cytotoxicity.

Following incubation, all samples were washed and analyzed using an Attune™ flow cytometer (Thermo Fisher Scientific (Waltham, MA, USA). Data acquisition and analysis were performed using FlowJo software (v10.10, Becton, Dickinson and Company (BD), Ashland, Oregon, USA).

### 4.18. Lactate Dehydrogenase (LDH) Cytotoxicity Assay

The CyQUANT™ LDH Cytotoxicity Assay (Thermo Fisher Scientific) was used to assess the cytotoxic activity of activated T cells against HCT-116 and HT-29 cell lines at effector-to-target (E:T) ratios of 10:1, 5:1, and 2:1. No non-tumorigenic or irrelevant target cell line was included as a control for non-specific cytotoxicity in the LDH assays.

Cancer cells were counted and seeded into 96-well plates at 100 μL per well in McCoy’s 5A medium (supplemented with 10% FBS and 1% streptomycin-penicillin) to allow for adherence. After 24 h of incubation, 50 μL of the medium was removed from each well. Activated T cells were then counted and added to the cancer cells at the indicated E:T ratios in 50 μL of RPMI-1640 medium (supplemented with 10% FBS and 1% streptomycin-penicillin). The co-culture plates were incubated for 24 h at 37 °C in a humidified atmosphere with 5% CO_2_. Following the incubation, 50 μL of supernatant from each well was transferred to a new 96-well plate. Then, 50 μL of a 1:1 mixture of Substrate Mix and Assay Buffer was added to each well containing the collected supernatant. After 30 min of incubation protected from light, 50 μL of Stop Solution was added to terminate the reaction. The plate was read at 490 nm (to measure LDH activity) and 680 nm (to account for background noise).

Percent cytotoxicity was calculated using the following formula: % Cytotoxicity = [(Experimental LDH release − Spontaneous release − Effector background)/(Maximum release − Spontaneous release)] × 100, where experimental refers to co-culture well, spontaneous to cancer cells alone, effector background = T cells alone and maximum release to cancer cells with lysis buffer.

### 4.19. RNA Isolation, cDNA Synthesis, and Quantitative Polymerase Chain Reaction (qPCR)

HCT-116, HT-29, and T cells were prepared as previously described. For co-culture experiments, CRC cells were seeded and allowed to adhere for 24 h, after which T cells were added at an E:T ratio of 10:1. Following the incubation period, adherent cells were collected for RNA isolation to specifically analyze *GZMA* and *PRF1* gene expression. RNA was isolated from adherent co-culture wells without prior physical separation of tumor and immune cell populations.

Total RNA was extracted using TRIzol Reagent (Invitrogen, Toulouse, France). After 24 h of incubation, the culture medium was removed, and 1 mL of TRIzol was added to lyse the cells, followed by incubation at room temperature for 5 min. Subsequently, 0.2 mL of chloroform was added, and after 3 min of incubation at room temperature, the samples were centrifuged at 12,000× *g* for 15 min at 4 °C. The mixture separated into three phases: a lower red organic phase containing proteins, a white interphase containing DNA, and an upper aqueous phase containing RNA. The upper aqueous phase was carefully transferred to a new tube, and 0.5 mL of isopropanol was added to precipitate RNA. After incubation at room temperature for 10 min, RNA was pelleted by centrifugation at 12,000× *g* for 10 min at 4 °C. The RNA pellet was washed with 70% ethanol, centrifuged at 7500× *g* for 5 min at 4 °C, air-dried for 3 min, and dissolved in 60 μL of DNase/RNase-free water. RNA concentration and purity were assessed using a spectrophotometer (Konica Minolta, Marconibaan, Netherlands).

cDNA was synthesized from 1 μg of total RNA using a reverse transcription kit according to the manufacturer’s instructions (RevertAid RT, Thermo Scientific, Waltham, Massachusetts, USA). RNA was combined with random hexamers, dNTPs, and nuclease-free water and heated at 65 °C for 5 min, then immediately chilled on ice for 5 min. Subsequently, reverse transcriptase, RNase inhibitor, and reaction buffer were added, and reverse transcription was performed at 30 °C for 10 min, followed by 42 °C for 30 min and 70 °C for 15 min. The synthesized cDNA was stored at 4 °C until used in quantitative PCR analysis.

qPCR was performed to quantify the mRNA expression levels of GZMA and PRF1 using SYBR Green (KAPA SYBR FAST Universal Kit, Sigma-Aldrich, Athens, Greece). Reactions were carried out in 96-well plates with a total volume of 10 μL per well, containing SYBR Green master mix, cDNA, primer mix (forward and reverse), and nuclease-free water. The primer sequences used were: GZMA forward: 5′-AGCAGTGGGAGACAGATGGA-3′ and reverse: 5′-TGGGAGGTTCTGAGGTTGAA-3′, PRF1 forward: 5′-TCTGCTCAGGAGTACCGTGA-3′ and reverse: 5′-AGGAGGTCGTTGTTGGTGTT-3′, GAPDH forward: 5′-TTGGTATCGTGGAAGGACTCA-3′ and reverse: 5′-TGTCATCATATTTGGCAGGTTTT-3′ and β-actin forward: 5′-AGAGCTAGAGCTGCCTGAC-3′ and reverse: 5′-AGCACTGTGTTGGCGTACAG-3′. Each sample was analyzed in technical triplicates, and all experiments were performed in three independent biological replicates. PCR cycling conditions consisted of initial denaturation at 95 °C for 3 min, followed by 35 cycles of 95 °C for 10 s, 60 °C for 30 s, and 72 °C for 30 s. Relative mRNA expression levels were calculated using the 2^−ΔΔCt^ method, normalized to GAPDH and β-actin, and expressed relative to the corresponding control cells cultured alone.

## 5. Conclusions

In conclusion, this study demonstrates that immune CYT is associated with immune infiltration patterns, immune checkpoint expression, and genomic features that collectively reflect the immune contexture of colorectal cancer. Rather than serving as a prognostic or predictive biomarker, CYT should be interpreted as a descriptive, integrative transcriptomic metric capturing aspects of cytotoxic immune activation within the TME.

Our findings highlight how CYT correlates with immune competency status, microsatellite instability, and tumor microenvironmental heterogeneity, while underscoring the complexity of immune regulation in colorectal cancer. Given the correlative nature of the analyses and the interpretive limitations of the functional assays, further longitudinal, multivariate, and mechanistic studies will be required to establish whether CYT has independent clinical or therapeutic relevance.

## Data Availability

Genomic data were extracted from TCGA (https://portal.gdc.cancer.gov/, accessed on 15 November 2023).

## Supplementary Materials

The following supporting information can be downloaded at https://www.mdpi.com/article/10.3390/ijms27146180/s1.
