# Supplementary material for: Immune Cytolytic Activity Correlates with Tumor Microenvironmental Aberrations in Colorectal Cancer
Source: Int J Mol Sci. 2026 Jul 10;27(14):6180. doi: 10.3390/ijms27146180 (PMC13409973; doi:10.3390/ijms27146180)
Supplement: Supplementary file 1 [file ijms-27-06180-s001.zip › ijms-4279781-supplementary/Supplementary Figures.pdf]

Supplementary Figures

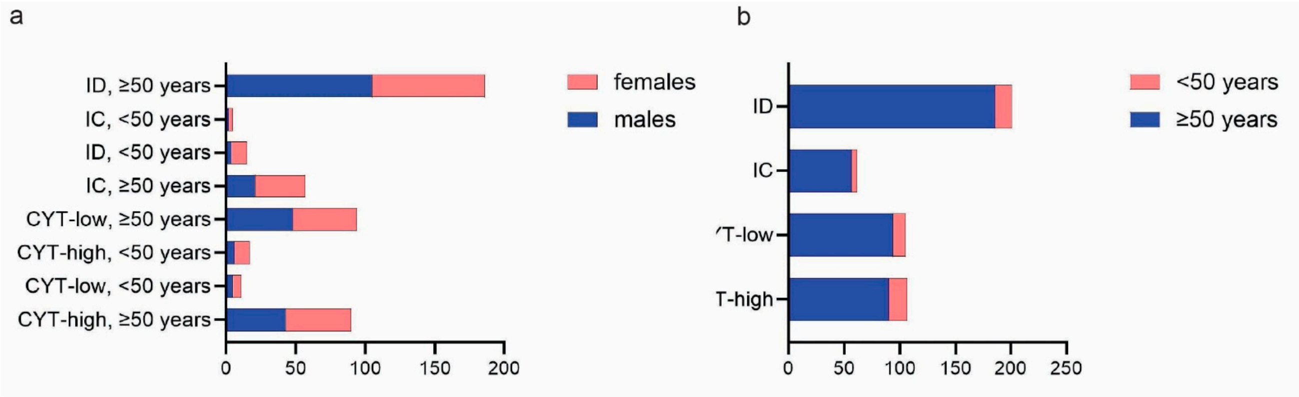

Supplementary Figure S1. (a) Distribution of cytolytic and immune-competency subgroups according to age and sex. Stacked bar plots show the number of male (blue) and female (salmon) patients stratified by age at diagnosis (<50 years and ≥50 years) across cytolytic activity (CYT-high and CYT-low) and immune-competency (immune-competent, IC; immune-deficient, ID) subgroups in the TCGA colorectal cancer cohort. Across all immune and cytolytic categories, the majority of tumors were diagnosed in patients ≥50 years of age, with relatively few early-onset cases. No clear age-associated enrichment of CYT-high versus CYT-low or IC versus ID tumors was observed. (b) Distribution of cytolytic activity and immune-competency subgroups according to age at diagnosis. Stacked bar plots show the number of colorectal cancer patients diagnosed at <50 years (salmon) and ≥50 years (blue) stratified by immune-deficient (ID) and immune-competent (IC) status, as well as by cytolytic activity (CYT-high and CYT-low). Across all immune and cytolytic categories, the majority of tumors occurred in patients ≥50 years of age. No clear age-associated differences in CYT or immune-competency classification were observed, although interpretation is limited by the small number of early-onset cases in the TCGA cohort.

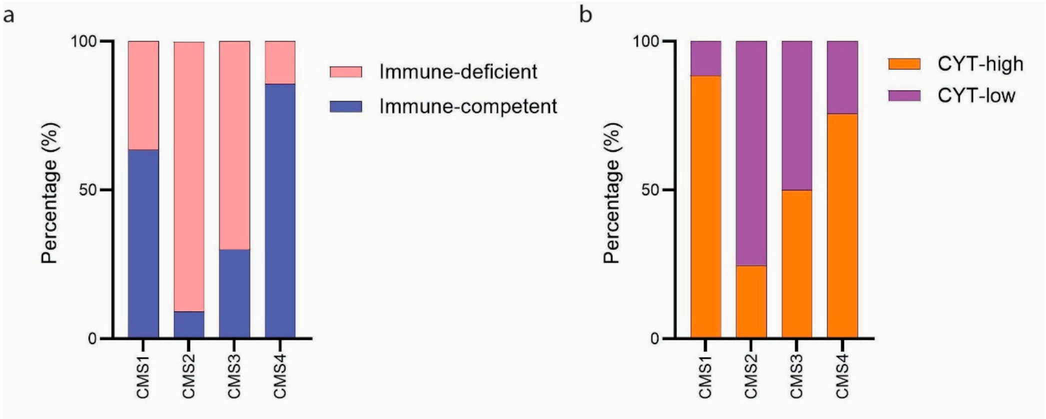

Supplementary Figure S2. (a) Distribution of Immune-competent (IC) vs Immune-deficient (ID) tumors across the different Consensus Molecular Subtypes (CMS1–4). The CMS subtype for each sample was extracted using the CMS classification pipeline, as in Guinney et al (2015). Clearly, the majority of CMS1 tumors are immune-competent, but still mixed. CMS2 and CMS3 tumors are strongly skewed toward immune-deficient; whereas CMS4 tumors are predominantly immune-competent. (b) Distribution of CYT-high vs CYT-low tumors across the different CMS subtypes. Clearly, the majority of CMS1 and CMS4 tumors are mainly CYT-high. CMS2 tumors are strongly skewed toward CYT-low; whereas CMS3 tumors are mixed.

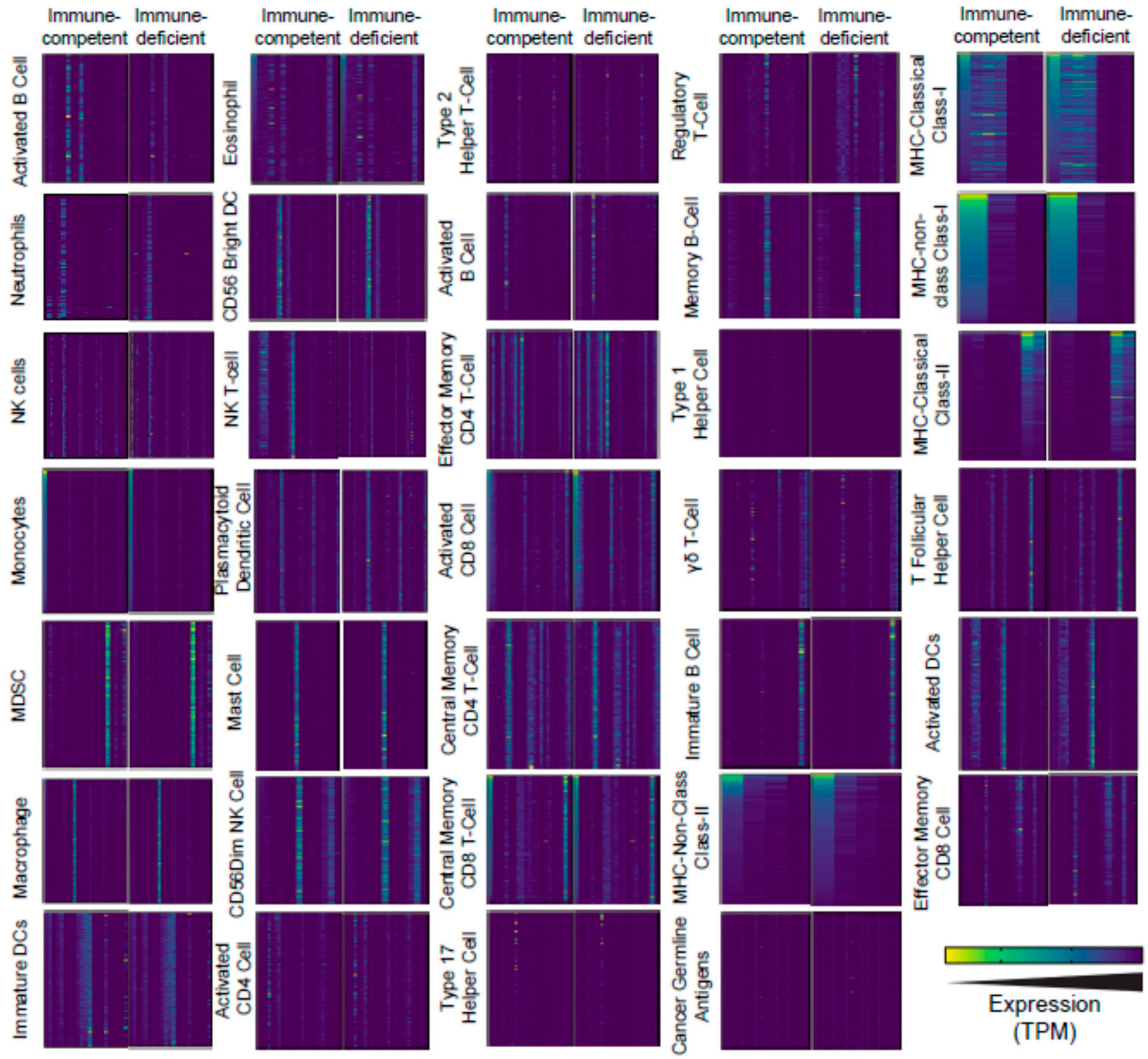

Supplementary Figure S3. Viridis colormaps depict various immune-related gene signatures in immune competent (IC) and immune-deficient (ID) tumors. Tumor samples are shown along the x-axis, while genes comprising each immune signature are displayed along the y-axis. Color intensity represents normalized gene expression values ($\log_2$[TPM + 1]), with darker colors indicating higher expression. Panels correspond to selected immune cell signatures relevant to cytotoxic and adaptive immune responses.

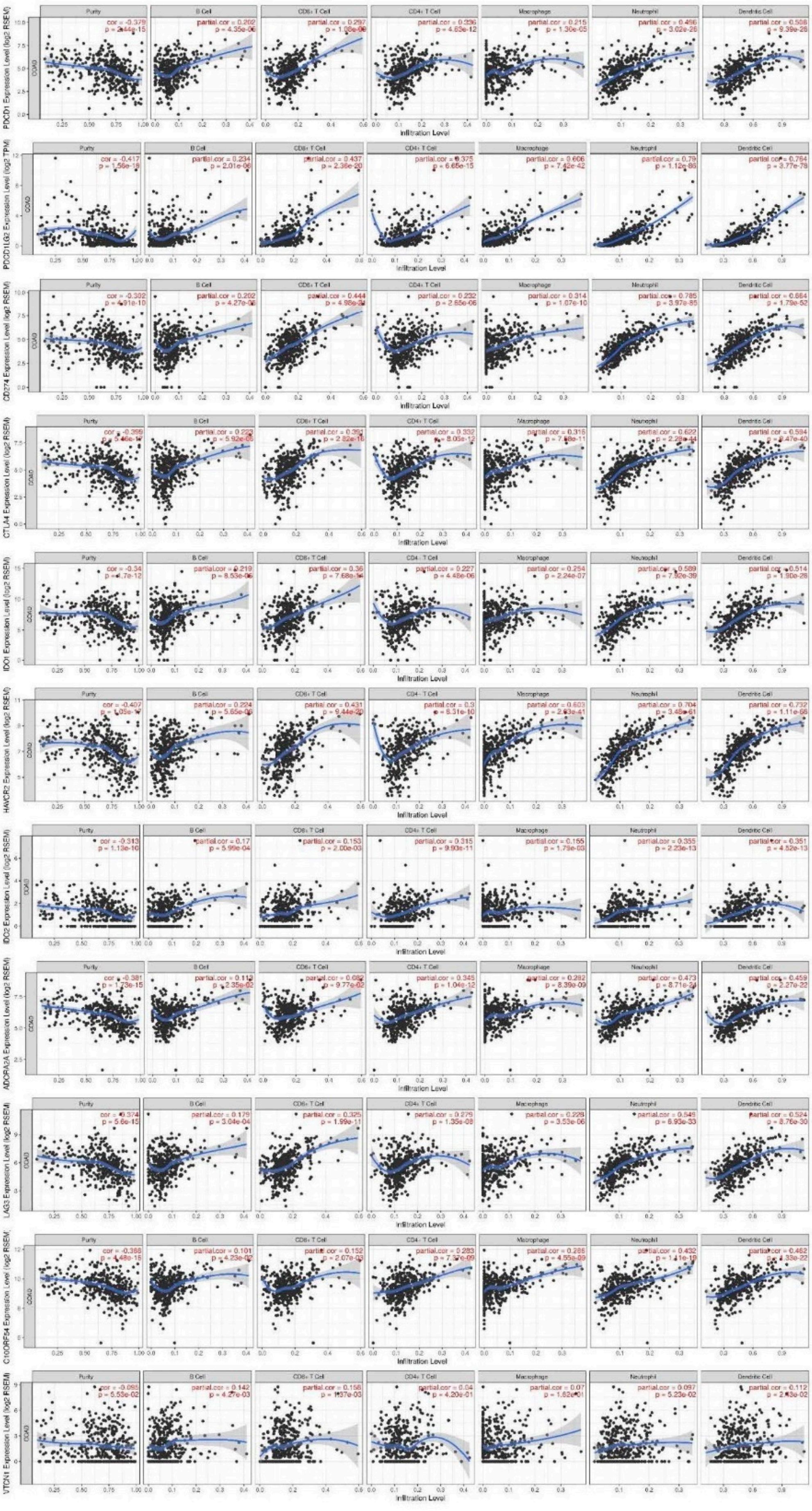

Supplementary Figure S4. Correlation between the expression of *PDCD1, CD274, PDCD1LG2, CTLA-4, IDO1, IDO2, HAVCR2, ADORA2A, LAG3, VISTA* and *VTCN1* (log_2RSEM) and infiltration of B cells, CD8+ T cells, CD4+ T cells, macrophages, neutrophils

and DCs in CRC. Spearman's correlation (rho values) and statistical significance (p-values) are depicted in each scatter plot.

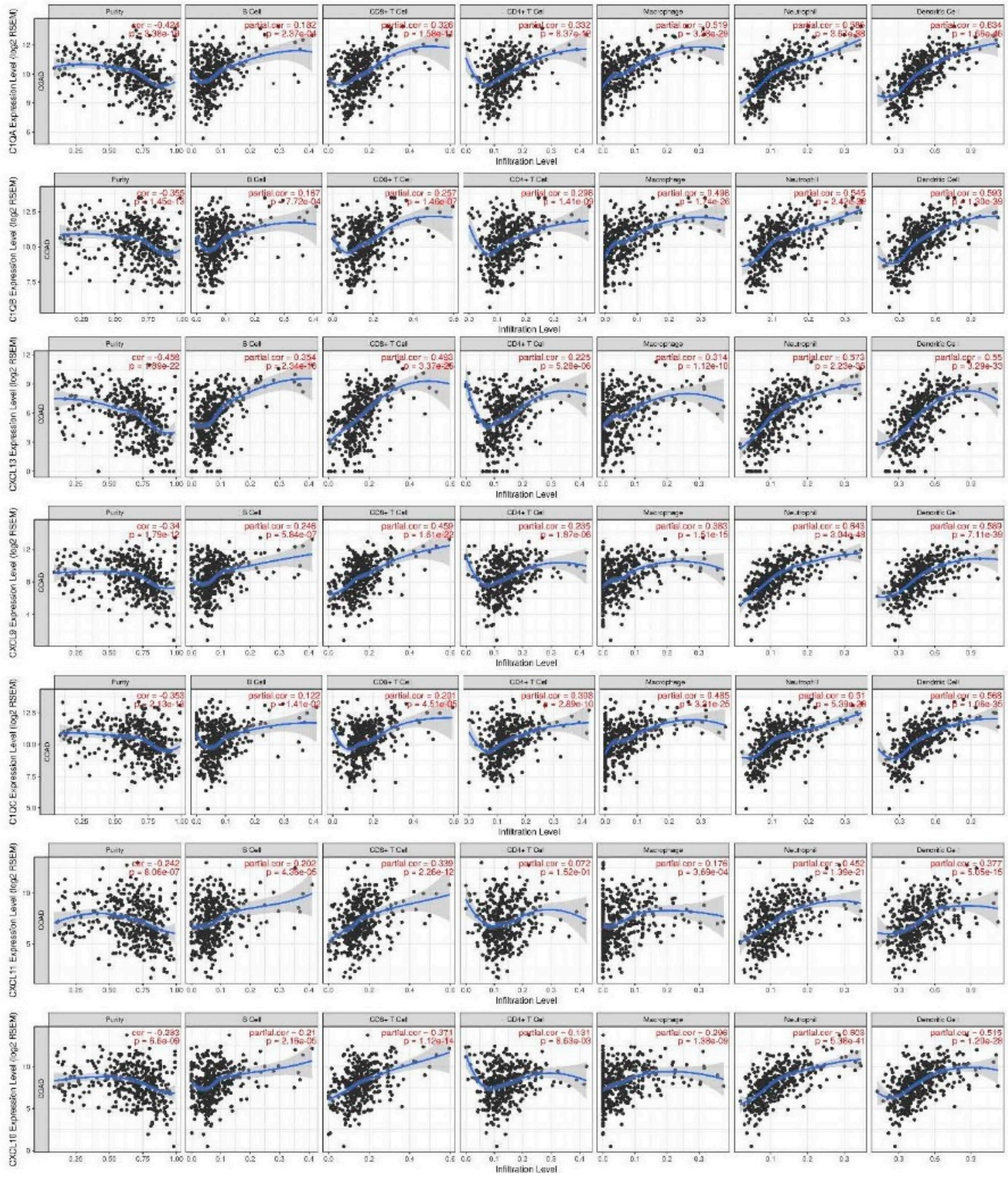

Supplementary Figure S5. Correlation between the expression of *C1QA, C1QB, C1QC, CXCL9, CXCL10, CXCL11* and *CXCL13* (log_2RSEM) and infiltration of B cells, CD8+ T cells, CD4+ T cells, macrophages, neutrophils and DCs in COAD. Spearman's correlation (rho values) and statistical significance (p-values) are depicted in each scatter plot.

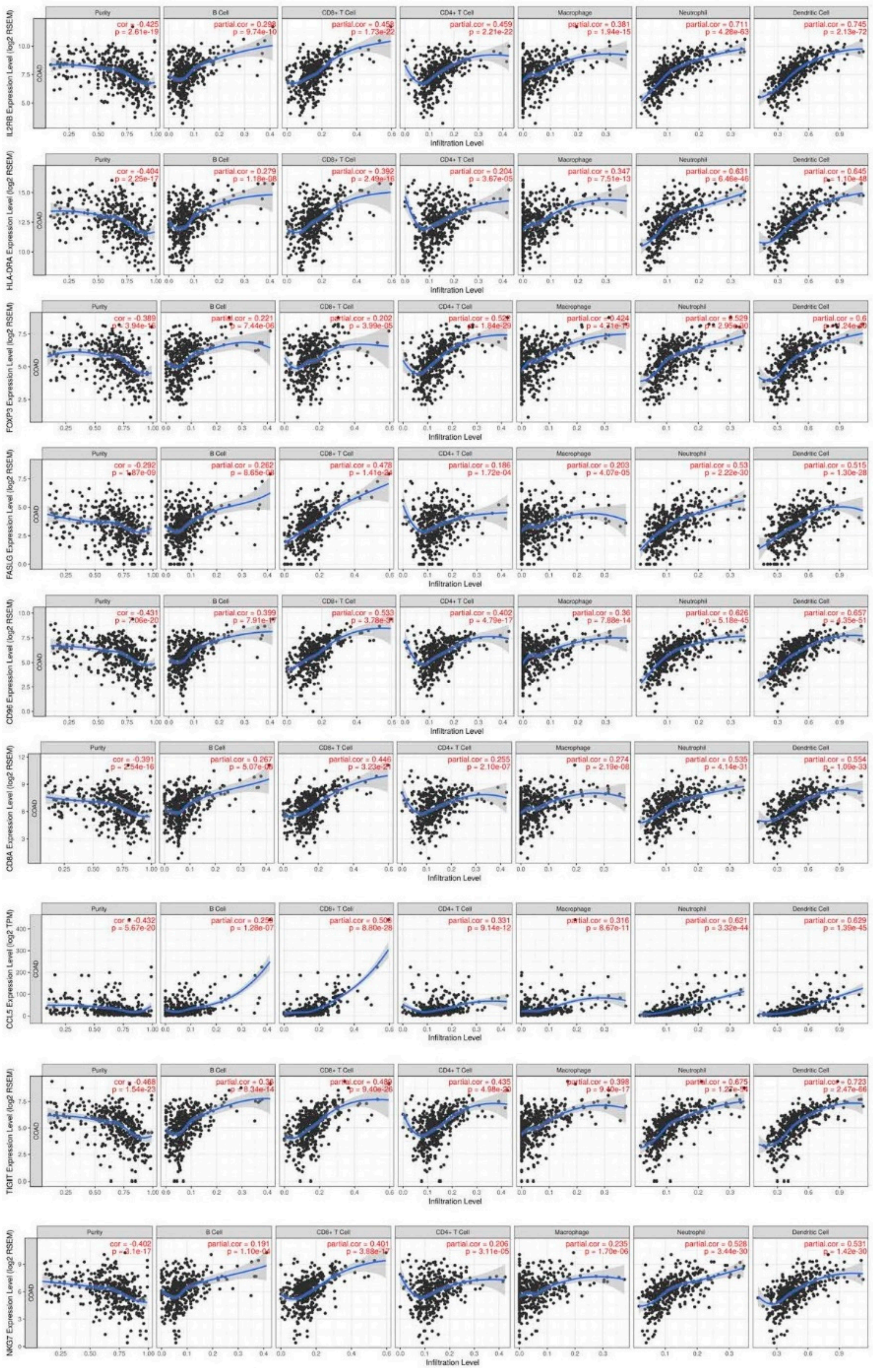

Supplementary Figure S6. Correlation between the expression of *IL2RB, TIGIT, CCL5, CD96, HLA-DRA, CD8A, GZMH, FASLG, NKG7* and *FOXP3* (log_2RSEM) and the infiltration of B cells, CD8+ T cells, CD4+ T cells, macrophages, neutrophils and DCs in CRC. Spearman's correlation (rho values) and statistical significance (p-values) are depicted in each scatter plot.
